# High Frequency Multi-Year Variability in Baltic Sea Microbial Plankton Stocks and Activities

**DOI:** 10.3389/fmicb.2018.03296

**Published:** 2019-01-17

**Authors:** Carina Bunse, Stina Israelsson, Federico Baltar, Mireia Bertos-Fortis, Emil Fridolfsson, Catherine Legrand, Elin Lindehoff, Markus V. Lindh, Sandra Martínez-García, Jarone Pinhassi

**Affiliations:** Centre for Ecology and Evolution in Microbial Model Systems – EEMiS, Linnaeus University, Kalmar, Sweden

**Keywords:** marine bacteria, phytoplankton, cyanobacteria, production, substrate uptake, enzyme activity, biogeochemistry

## Abstract

Marine bacterioplankton are essential in global nutrient cycling and organic matter turnover. Time-series analyses, often at monthly sampling frequencies, have established the paramount role of abiotic and biotic variables in structuring bacterioplankton communities and productivities. However, fine-scale seasonal microbial activities, and underlying biological principles, are not fully understood. We report results from four consecutive years of high-frequency time-series sampling in the Baltic Proper. Pronounced temporal dynamics in most investigated microbial variables were observed, including bacterial heterotrophic production, plankton biomass, extracellular enzyme activities, substrate uptake rate constants of glucose, pyruvate, acetate, amino acids, and leucine, as well as nutrient limitation bioassays. Spring blooms consisting of diatoms and dinoflagellates were followed by elevated bacterial heterotrophic production and abundances. During summer, bacterial productivity estimates increased even further, coinciding with an initial cyanobacterial bloom in early July. However, bacterial abundances only increased following a second cyanobacterial bloom, peaking in August. Uptake rate constants for the different measured carbon compounds varied seasonally and inter-annually and were highly correlated to bacterial productivity estimates, temperature, and cyanobacterial abundances. Further, we detected nutrient limitation in response to environmental conditions in a multitude of microbial variables, such as elevated productivities in nutrient bioassays, changes in enzymatic activities, or substrate preferences. Variations among biotic variables often occurred on time scales of days to a few weeks, yet often spanning several sampling occasions. Such dynamics might not have been captured by sampling at monthly intervals, as compared to more predictable transitions in abiotic variables such as temperature or nutrient concentrations. Our study indicates that high resolution analyses of microbial biomass and productivity parameters can help out in the development of biogeochemical and food web models disentangling the microbial black box.

## Introduction

In many aquatic environments, microbially mediated fluxes of energy and matter change dynamically over the year, alongside seasonal variation in community structure and genetic make-up of marine bacterial assemblages (Fuhrman et al., 2006; Gilbert et al., 2012; Teeling et al., 2012; Hugerth et al., 2015; Lindh et al., 2015; Teeling et al., 2016; Bunse and Pinhassi, 2017). This shows that bacterial assemblages can systematically respond to environmental cues, both in species composition and metabolic activity, facilitating interactions with the environment. River runoff and phytoplankton photosynthesis (i.e., primary production) are major sources of dissolved organic matter (DOM) for marine heterotrophic bacteria (Cole et al., 1988; Baines and Pace, 1991). At a global scale, it is in fact estimated that bacterial assemblages assimilate, respire, and metabolize around half of the carbon fixed through primary production (Azam et al., 1983; Azam, 1998). Yet, detailed knowledge is necessary on the spatiotemporal variability in linkages between environmental conditions and bacterial community functional adaptations at relevant temporal scales (days to years), i.e., the metabolic plasticity of bacteria in terms of heterotrophic activity, to obtain an understanding of how aquatic microbes ultimately influence fluxes of DOM, molecules, and nutrients essential for life.

Phytoplankton biomass and net primary production in temperate regions change with seasons (Behrenfeld et al., 2005; Tiselius et al., 2015). In the Baltic Proper, the spring bloom is comprised of mainly diatoms and dinoflagellates, but in recent decades a trend toward decreasing diatom biomass and increasing dinoflagellate dominance has been reported (Klais et al., 2011; Wasmund et al., 2011; Legrand et al., 2015). During summer, diazotroph filamentous cyanobacteria [mainly *Aphanizomenon* sp., *Dolichospermum* sp. (previously defined as *Anabaena* sp.), and *Nodularia spumigena*] thrive and form large blooms in the Baltic Proper (Bianchi et al., 2000; Wasmund and Uhlig, 2003; Thamm et al., 2004; Suikkanen et al., 2007; Bertos-Fortis et al., 2016). Also protists (often referred to as pico- or nanoeukaryotes) in the Baltic Sea are reported to undergo changes in seasonal succession (Samuelsson et al., 2006). Therefore, it is paramount to unravel short-term, inter-, and intra-annual phytoplankton dynamics (i.e., changes in magnitude and composition) further, and how such variability affects ecosystem functions at both higher and lower trophic levels.

Oceanic time-series are essential for understanding marine processes since they provide knowledge of marine habitat variability, biogeochemical overturn, regulation of planktonic processes, and climate trends worldwide (Karl and Lukas, 1996; Tiselius et al., 2015). Using multidisciplinary approaches, insights into local microbial food webs and natural ecosystem variability can be gained (Karl and Lukas, 1996). In the semi-enclosed Baltic Sea, monitoring stations have been established in most basins to estimate and monitor the eutrophication status and phytoplankton dynamics of the Baltic Sea following reports of the Helsinki Commission [for example analyzed in Wasmund and Uhlig (2003), Suikkanen et al. (2007), and Backer et al. (2010)]. However, data on several important microbial variables, such as bacterial abundances, bacterial community composition, bacterial heterotrophic production, and other activity estimates, are strikingly missing in Baltic Sea datasets, with a few notable exceptions (Hoppe et al., 2013; Huseby and Wikner, 2015; Lindh et al., 2015). Further, even less information about the linkages between phytoplankton and bacteria from the Baltic Sea are available at high resolution. Given the importance of microbial communities for ecosystem productivity and nutrient (re)cycling, time-series investigating variability in bacterioplankton productivity linked to ecosystem dynamics (on time-scales revealing cues to the factors regulating microbial activity) are imperative.

Here we present high-frequency data of abiotic and biotic parameters from the Linnaeus Microbial Observatory (LMO), situated in the Western Baltic Proper, during the period 2011–2014. Several reports have presented data subsets from this site or shown low frequency data of environmental, microbial, or genetic data (metabarcoding or metagenomics) from 2011 to 2013 (Hugerth et al., 2015; Legrand et al., 2015; Baltar et al., 2016; Bertos-Fortis et al., 2016; Lindh et al., 2017; Alneberg et al., 2018a,b; Paerl et al., 2018), yet see the 2011 high frequency study (Lindh et al., 2015). For a full presentation of the LMO sampling site, please also see: https://lnu.se/en/research/searchresearch/linnaeus-microbial-observatory-lmo/. Here we profoundly extend the analysis of the microbial food web at LMO and present new nutrient limitation bioassays and substrate uptake rate constants. The aim of the study was to examine seasonal fluctuations in bacterial heterotrophic activities (bacterial heterotrophic production, extracellular enzymatic activities, and substrate uptake rate constants) in relation to phytoplankton community dynamics and changes in physicochemical conditions at a multi-year high-resolution temporal scale – an unprecedented effort for the Baltic Sea. We hypothesized that an elevated sampling frequency (twice weekly to bi-weekly or monthly), as compared to typical monitoring schemes (monthly or longer), would allow analyses of temporal variability in amplitude of microbial processes, and more firmly establishing linkages between such processes and environmental conditions and phytoplankton dynamics. This, in turn, would allow interpreting potential ecological drivers of more labor-intensive and less frequently sampled rate measurements and bacterial activities.

## Materials and Methods

### Field Sampling and Location

**FIGURE 1:**
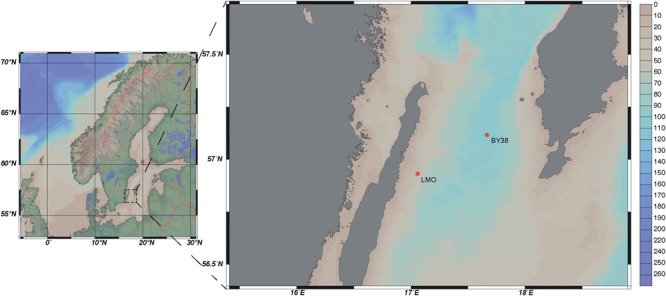
Bathypelagic map of LMO and SMHI monitoring station KARLSÖDJ (BY38) (Ocean Data View, ODV), depth profile in meters.

The LMO sampling site is located 11 km off the northeast coast of Öland in the Western Gotland Sea (N 56°55.8540′, E 17°3.6420′), belonging to the Baltic Proper. LMO is situated between SMHI/HELCOM monitoring stations BY38 and BY39 and further details on the sampling site are described in Lindh (2014) and Legrand et al. (2015). Seawater was collected nearly twice weekly during 2011–2013 and monthly during 2014. Water from 2 m depth was sampled using 3 or 5 l Ruttner water samplers at ∼9 a.m. during each sampling occasion (Figure 1). Water was collected into 10 l acid-washed polycarbonate bottles and transported to the laboratory within ∼1 h. Upon arrival to the laboratory, samples for chlorophyll *a* (Chl *a*), nutrients, phytoplankton biomass and composition, bacterial abundance, and heterotrophic production were collected. Extracellular enzyme activity, substrate uptake rates, and nutrient limitation samples were taken from the seawater transported to the laboratory every second week during 2012–2014.

### Abiotic Parameters

Temperature and salinity were measured on site. In addition, temperature, salinity, and light profiles in the whole 40 m deep water column were sampled using a CTD probe (AAQ 1186-H, Alec Electronics, Japan) since 2013. Samples for total nitrogen (TN) and dissolved organic carbon (DOC) were collected in duplicates; 20 ml seawater were filtered through precombusted (450°C, 2 h) GF/C glass fiber filters via gravity and stored in precombusted tubes with 200 μl HCl (2 M) added, at 6–8°C until analysis. DOC and TN were measured via high temperature catalytic oxidation (HTCO) using a Shimadzu TOC-V analyzer coupled to a TNM-1 unit (2011–2013) and DOC was further analyzed by a Shimadzu TOC-L Total Organic Carbon Analyzer (Shimadzu Corporation) during 2014 (Traving et al., 2017). The detection limit for DOC using these instruments were at or below 0.13 mg l^-1^ and the detection limit for TN is at or below 5 μg l^-1^ (company product information and personal communication with H. Larsson, Umeå Marine Sciences Centre). The 2011 DOC data were excluded from further analysis due to DOC contamination during that year. We further identified two samples (2014-05-06; 682 μM and 2014-11-20; 680 μM) as outliers which were removed in the graphs and statistical analyses [Bonferroni Outlier test (*outlierTest*) (Fox and Weisberg, 2011)]. Averages of duplicates are presented. Dissolved inorganic nutrients [${\mathrm{NO}}_{3}^{–}$ and ${\mathrm{NO}}_{2}^{–}$ (together presented as nitrate, ${\mathrm{NO}}_{3}^{–}$), ${\mathrm{NH}}_{4}^{+}$, ${\mathrm{PO}}_{4}^{3–}$, and SiO_2_] and total phosphorus (TP) were collected and frozen until analysis using colorimetric methods (UV-1600 Spectrometer, VWR) (Valderrama, 1995).

### Biotic Parameters

Chlorophyll *a* was extracted and measured following the Jespersen and Christoffersen (1987) protocol. Briefly, 500 ml seawater was filtered on (A/E) glass fiber filters (∼1 μm pore size) under low vacuum and extracted in ethanol (96%) in darkness. These filters catch single-celled cyanobacteria down to the size of *Synechococcus* that are abundant in the Baltic Sea; *Prochlorococcus* are not found in the Baltic Sea and *Cyanobium* only at times account for minor portions of the Chl *a* (Komárek et al., 1999; Haverkamp et al., 2009; Bertos-Fortis et al., 2016; Celepli et al., 2017). Chl *a* concentrations were measured using a Turner fluorometer, average values of technical duplicates are presented. Samples for phytoplankton community composition were collected and counted microscopically after preservation with acid Lugol’s solution following (Utermöhl, 1931, 1958; Legrand et al., 2015). Briefly, 500 ml sample for phytoplankton abundance were fixed using 2% Lugol’s solution and kept in darkness until processing. Two to ten milliliters of sample were subsequently transferred into sedimentation chambers and counted using an Olympus CK X41 microscope. For each sample, a minimum of 300 cells were counted and identified to genus or species level when possible. Phytoplankton biomass was calculated based on biovolume (Olenina et al., 2006) and carbon content (Edler, 1979).

Bacterial abundance was preserved in duplicates with formaldehyde as described in Lindh et al. (2015) and enumerated in a flow cytometer (Facs Calibur Becton Dickinson in 2011–2012 and Partec Cube8 in 2013–2014) as described in Giorgio et al. (1996). Bulk bacterial abundance samples were stained with SYTO13 (Life Technologies) during 2011–2012 and SYBR Green during 2013–2014 (Life Technologies). Briefly, cells were thawed in darkness at room temperature, stained using SYTO13 or SYBR Green (5 μM final concentration) and incubated in darkness for 15 min, before enumeration. Averages of technical duplicates are presented.

For bacterial heterotrophic production estimates, we diluted commercial ^3^H-leucine (Perkin Elmer; 1 mCi ml^-1^) to 1 μM using cold leucine (Gasol et al., 1998). Then the samples were incubated with lukewarm ^3^H-leucine (40 nM final concentration) for 2 h at approximate *in situ* temperatures in at least triplicates with one killed control [trichloric acid (TCA); 5% final concentration; Sigma-Aldrich] following the leucine incorporation and centrifugation protocol described in Smith and Azam (1992). We assumed a conversion factor of 0.86 for the transformation from cellular carbon to protein, a factor of 0.073% leucine in total proteins and an isotope dilution of 2 according to Simon and Azam (1989) as presented in previous LMO reports [see for example Lindh et al. (2015) and Baltar et al. (2016)]. Average values of technical replicates are presented.

Extracellular enzymatic activity and substrate uptake rate constants (*K*) were determined biweekly from March 2012 to December 2013, and monthly during 2014. Extracellular enzymatic activities; β-glucosidase, leucine aminopeptidase (LAPase), and alkaline phosphatase (APase) were determined in technical quadruplicates according to the fluorometric enzyme assays and conditions described in Baltar et al. (2016). Substrate uptake rate constants (*K*) were determined in technical triplicate 10 or 30 ml samples with one formaldehyde killed control (Sigma-Aldrich; 1.4% final concentration) using ^3^H labeled L-leucine and glucose (final concentrations 0.5 and 1.0 nM, respectively) and ^14^C labeled L-amino acid mix (dissolved free amino acids, DFAA), acetate, and pyruvate (final concentrations 1.0, 10, and 10 nM, respectively, PerkinElmer). Samples were incubated in the dark at *in situ* temperature for 1 h (a linear relationship between *K* and incubation time ranging between 30 min and 3 h was determined) and killed by adding formaldehyde (Sigma-Aldrich; 1.4% final concentration) 10 min prior to filtration through 0.2 μm polycarbonate filters (25 mm diameter, GVS Life Sciences). Filters were rinsed with 2 ml 0.9 M NaCl three times and placed in scintillation vials. Three milliliters of Ultima Gold^TM^ XR liquid scintillation cocktail (Sigma) was added, samples were incubated in the dark for 18 h and counted in a liquid scintillation counter (Wallac WinSpectral 1414). Disintegrations per minute (DPM) of negative controls were subtracted from sample mean DPM and *K* was calculated for mean DPM using Equation (1) according to a first-order reaction type, where *A* = total radioactivity added (DPM ml^-1^), *a* = incorporated radioactivity (DPM ml^-1^), and *t* = time (h). Hence, *K* illustrates the substrate uptake rate constant of a specific substrate over time.

(1) $$\begin{array}{c} K=(-\ln((A-a)/A))/t \end{array}$$

To investigate the potential of limiting nutrients (C glucose, N ammonium, P phosphate) for bacterial growth, we performed nutrient limitation assays. Bacterial nutrient limitation was determined by aliquoting 250 ml of seawater to acid washed and Milli-Q rinsed polycarbonate bottles and adding 24 μM carbon (C) as glucose, 4.2 μM nitrogen (N) as ammonium (NH_4_Cl), and/or 0.6 μM phosphorus (P) as phosphate (NaH_2_PO_4_). As in previous experiments (Pinhassi et al., 2006), these additions were chosen since heterotrophic bacteria typically have lower C:N:P ratios than phytoplankton, e.g., 45:10:1 for bacteria in the Bothnian Sea (Zweifel et al., 1993) or see ratios reported by Fagerbakke et al. (1996) from a broad variety of environmental and laboratory settings (Fagerbakke et al., 1996). Nutrients were added in duplicate (C, N, P, and CP) or single (CN, NP, and CNP) treatments compared to control treatments (*K*) and incubated in the dark for 24 h at 16°C 2012 until summer 2013, after summer 2013 bottles were incubated at approximate *in situ* temperatures. After 24 h, differential responses to nutrient addition were determined by measuring bacterial heterotrophic production as described above. Optimally, these nutrient enrichment bioassays were done in the dark as to measure the short-term response of the heterotrophic bacteria to the specific experimental changes in nutrient availability. However, in reality, multiple biological activities that are associated with diurnal variations in light are likely to influence the heterotrophic bacterial production also in such dark incubations, like changes in production/consumption of DOC by phytoplankton or mixotrophic flagellates. The precise influence of such factors on measures of short-term bacterial activities remains unknown, but the minor changes in the controls compared to the cases where enrichments have an effect indicates that, overall, the bioassay approach provides interpretable results. We consider a response in bacterial heterotrophic production to nutrient additions, in comparison to control incubations, as a proxy for growth limitation and the results are presented as bacterial heterotrophic production subtracted by the bacterial heterotrophic production of the control treatment.

### Statistical Analysis and Graphical Outputs

Seasons in the Baltic Proper were defined according to HELCOM as follows: Spring: March–May, Summer: June–September, Autumn: October–December, and Winter: January–February (Wasmund et al., 2011). Standard deviations for technical replicates (triplicates up to quadruplicates) were determined using the *n-*1 method. Correlations between variables were determined using Spearman’s rank correlation tests as data were non-normally distributed (*n* = 124 after omitting NAs). For the Spearman’s rank correlation tests of the DOC data, 2011 data and the two outliers were removed prior correlation analysis and results are presented in a separate figure (Supplementary Figure 3). For the Spearman’s rank correlation tests of the substrate uptake rate constants, data were subsampled to the bi-weekly sampling scheme (*n* = 38) prior correlation analysis. All correlations were considered significant when *ρ* > 0.25 and *p*-value after Bonferroni correction resulted in *p* < 0.05. Differential nutrient limitation, enzyme activities, and substrate uptake rate constants between years, seasons, and substrates/enzymes were tested using Monte Carlo simulation randomization test (1000 permutations) and results were considered significant when the *p*-value after Bonferroni correction resulted in *p* < 0.05. All statistical tests were performed in RStudio [Version 1.1.453 –© 2009–2018 RStudio, Inc., R version 3.3.3 GUI 1.69 Mavericks build (7328)] using the vegan package (Oksanen et al., 2007). Figure 1 was drawn using Ocean Data View. All other graphical outputs were made in RStudio (Version 1.1.453 –© 2009–2018 RStudio, Inc.) using the ggplot2 package (Wickham, 2009).

## Results

### Patterns in Environmental Conditions

**FIGURE 2:**
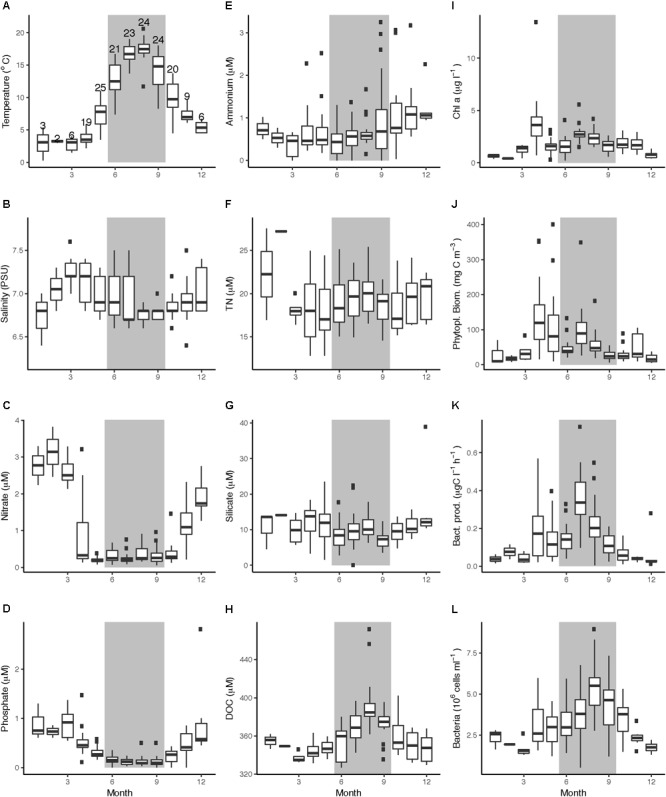
Boxplots of abiotic and biotic data at LMO over the period of 2011–2014, grouped by month. **(A)** temperature (°C), **(B)** salinity (PSU), **(C)** nitrate (μM), **(D)** phosphate (μM), **(E)** ammonium (μM), **(F)** TN (μM), **(G)** silicate (μM), **(H)** DOC, **(I)** Chl *a* (μg l^-1^), **(J)** phytoplankton biomass (mgC m^-3^), **(K)** bacterial heterotrophic production (μg C l^-1^ h^-1^), and **(L)** bacterial abundances (cells ml^-1^). Gray backgrounds indicate summer months. In panel **A**, the number of samplings per month over the 4-year time series is indicated above the boxplot (#*n*), note though that TN and DOC data comprise of less number of samplings.

Across the four studied years, temperature ranged from 0.3 to 5.4°C in Winter and increased during spring, with highest variability in May (3.5–11.0°C) and Summer (7.4–16.7°C) (Figure 2A). During July and August, temperatures were stable around 17°C and the water column was stratified with a mixed layer depth of approximately 15 m (data not shown). Temperature decreased with high variability during September (18.0–8.3°C) and October (13.7–4.5°C). Salinity ranged between 6.4 and 7.6 PSU with little inter-annual variation (Figure 2B). Upon deep water mixing in late autumn and early winter, nitrate concentrations increased to reach peak concentrations of ∼3.1 μM in February, and then rapidly decreased in early April to around or below 0.33 μM, with low concentrations until October (a reduction of >88%, Figure 2C). Phosphate concentrations in winter ranged 0.61–2.81 μM, and somewhat more slowly compared to nitrate, decreased during April and May to below 0.2 μM in summer (Figure 2D). Phosphate peaked in December 2011 (2.81 μM) and showed considerably higher concentrations during the subsequent spring of 2012 compared to the other years (Supplementary Figure 1). Ammonium concentrations were fairly stable <1.0 μM through the year, but over the productive seasons intermittent peaks reaching over 2 μM were observed (Figure 2E). Concentrations of TN averaged 18.9 μM during the study with a slight decrease in spring (Figure 2F) and accumulated over the productive season in 2012. Silicate concentrations decreased over the productive seasons of 2011 and 2013, from 13 to 1.6 μM and from 17 to 5 μM, respectively, but in 2012, two periods of intermittent peaks occurred, up to 23 μM (Figure 2G). DOC concentrations in 2012–2014 ranged between 327.0 and 471.8 μM (mean 360.7 μM) and increased by ∼50 μM between spring and summer (mean concentrations of 335.1 μM in March and 384.8 μM in August) followed by a decrease from September to November (Figure 2H). Among the physico-chemical parameters, Spearman’s rank correlation test over the 4 years showed that temperature was significantly negatively correlated with salinity and the nutrients phosphate and nitrate and positively correlated with DOC (Supplementary Figures 2, 3).

### Variation in Phytoplankton Species Composition and Biomass Amplitudes

**FIGURE 3:**
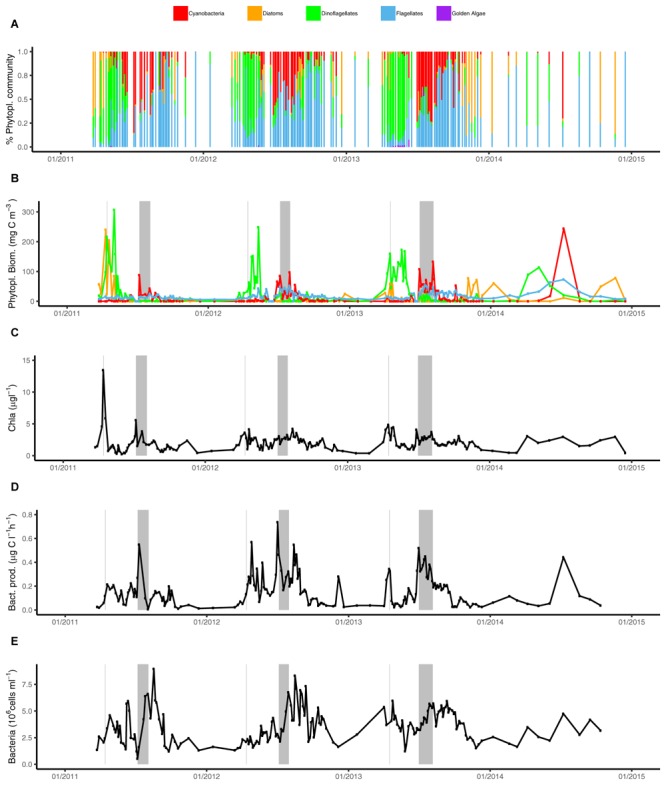
Phytoplankton community composition and heterotrophic bacterial heterotrophic production over time (2011–2014) at LMO. **(A)** Relative carbon contribution of phytoplankton groups to the total phytoplankton biomass; cyanobacteria (red), diatoms (orange/brown), dinoflagellates (green), flagellates (blue), and golden algae (pink); **(B)** phytoplankton biomass (mgC m^-3^) separated into major phyla: cyanobacteria (red), diatoms (orange), dinoflagellates (green), and flagellates (blue); **(C)** Chl *a* (μg l^-1^), **(D)** heterotrophic bacterial heterotrophic production (μg C l^-1^ h^-1^), and **(E)** bacterial abundances (cells ml^-1^). Gray background lines indicate Chl *a* spring bloom maximum and gray background boxes indicate the time between the two summer cyanobacteria maxima.

Chlorophyll *a* concentrations followed a seasonal pattern over the years with low concentrations in winter (<0.75 μg l^-1^) and elevated values in early April (average 3.82 μg l^-1^) and summer (July–August). The highest Chl *a* concentrations in the current study were detected during the 2011 spring bloom (13.45 μg l^-1^) (Figure 3C). During summer month (June–September), Chl *a* concentrations averaged 1.68 μg l^-1^ (2011), 2.41 μg l^-1^ (2012), 2.16 μg l^-1^ (2013), and 2.1 μg l^-1^ (2014) (Supplementary Table 1). There was a tendency toward another period of elevated Chl *a* in late autumn (October–November, 1.8 μg l^-1^) (Figures 2I, 3C). Phytoplankton biomass based on microscopy counts roughly followed Chl *a* concentrations; during the spring bloom, biomass averaged 138 mgC m^-3^ (up to 354 mgC m^-3^). Phytoplankton biomass remained high during May (average 106 mgC m^-3^) before it dropped in June (average 51 mgC m^-3^). During July, phytoplankton biomass increased again (average 101 mgC m^-3^, with peaks up to 349 mgC m^-3^), whereas autumn values remained below 105 mgC m^-3^ (Figure 2J).

Phytoplankton community composition varied strikingly across seasons (Figures 3A,B). Diatoms and dinoflagellates dominated the spring phytoplankton community prior to water column stratification. In 2011, the highest peak in diatom spring biomass was observed at 240 mgC m^-3^, while in the following years, spring peak diatom biomass was as much as 10 times lower. Irrespective of spring diatom biomass, diatoms showed elevated biomass during autumn and in winter. Notably, from 2012 to 2014, the highest annual diatom biomass concentrations were detected during autumn and/or winter (between 25 mgC m^-3^ in 2012 and 78 mgC m^-3^ in 2014). Maximum dinoflagellate biomass was consistently observed in spring at 114–307 mgC m^-3^, albeit with maximum biomass decreasing each year (2011–2014) (Figure 3B).

Filamentous cyanobacteria dominated the summer phytoplankton community all 4 years, reaching 88–244 mgC m^-3^, and bloomed under stratified conditions from June to September. The cyanobacterial blooms during 2011–2013 were characterized by an initial peak in the end of June/beginning of July, a slightly lower biomass in mid-July and a second peak in the end of July/beginning of August. For each successive year over the sampled period, the maximum biomass of cyanobacteria slightly increased, with a maximum of 244 mgC m^-3^ observed in 2014 (Figure 3B). Flagellates biomass averaged 18 mgC m^-3^ over the sampling period (from <1 to max = 73 mgC m^-3^), and displayed elevated biomass between May and October (reaching 50–73 mgC m^-3^ in summer), but in 2011 biomass reached only 25 mgC m^-3^. Despite the relatively low biomass during the year compared to dinoflagellates or cyanobacteria, flagellates frequently contributed >80% of total phytoplankton biomass during extended periods in autumn (Figure 3A).

### Bacterial Heterotrophic Production and Bacterial Abundances Over the Seasons

Bacterial heterotrophic production typically increased from low levels in autumn and winter (<0.06 μgC l^-1^ h^-1^) to a first peak averaging 0.18 μgC l^-1^ h^-1^ around mid-April following phytoplankton spring blooms (Figures 2K, 3D). A second and larger peak was observed in July averaging 0.36 μgC l^-1^ h^-1^. Bacterial heterotrophic production was generally higher during 2012, particularly in spring (following a strong dinoflagellate bloom). With the monthly sampling frequency in 2014, a spring peak in production was not detected. Spring peaks in bacterial heterotrophic production dropped down to baseline levels within 2 weeks and remained low until summer. The timing of the summer peaks in bacterial heterotrophic production (2011–2014) coincided with the increase of cyanobacterial biomass in the beginning of July (Figure 3D). Incidentally, as for cyanobacterial biomass, the 2011 summer peak was initiated ∼10 days later compared to subsequent years.

Bulk bacterioplankton cell numbers varied substantially over the 4 years, ranging between 0.5 and 8.9 × 10^6^ cells ml^-1^ (Figure 2L). Winter abundances over the 4 years were low (between 1.3 to 2.8 × 10^6^ cells ml^-1^) and cell abundance increases following the phytoplankton blooms were delayed with respect to bacterial heterotrophic production (Figure 3E). A slight dip in bacterial abundance followed the spring peak (around early June) before abundances increased toward summer. The highest variance in bacterial abundances was observed in July (range of 0.5–6.8 × 10^6^ cells ml^-1^) and August (2.8–8.9 × 10^6^ cells ml^-1^). The spring peak in 2012 was lower compared to the other years but summer bacterial abundance that year was high. Remarkably, during 2011–2013 there was a near-linear increase for approximately 10 days in bacterial abundances in late July, just prior to the second peak in cyanobacteria biomass, while bacterial heterotrophic production peaked during the first cyanobacteria biomass increase (Figures 3D,E).

Spearman’s rank correlation test showed significant correlations between bacterial heterotrophic production and abiotic (temperature, nitrate, phosphate) and biotic (Chl *a*, phytoplankton biomass, cyanobacterial biomass, flagellate biomass) parameters (Table 1). Bacterial abundances on the other hand, correlated significantly with temperature, salinity, phosphate, DOC, Chl *a*, cyanobacteria, and flagellate biomass (Table 1). To evaluate the influence of sampling frequency on the variance of bacterial and phytoplankton biomass production, samples were subsampled to monthly intervals (first week of the month). Thus, looking at the bacterial abundance, as well as Chl *a* or phytoplankton biomass at each first week of the month, a substantial amount of variability was lost (Supplementary Figure 4). Especially subsampling the 2012 data, spring phytoplankton bloom peak abundances and bacterial abundances were missed.

**Table 1:** Correlations of bacterial heterotrophic production (μg C l^-1^ h^-1^) and bacterial abundances (cells ml^-1^) against measured abiotic and biotic environmental data (Spearman’s rank correlation test, *n* = 124).

	Bacterial heterotrophic production (μg C l^-1^ h^-1^)		Bacterial abundance (cells ml^-1^)	
Variable	*ρ*	*p*-value	*ρ*	*p*-value
Temperature (°C)	0.54	<0.000001*	0.62	<0.000001*
Salinity (PSU)	–0.12	0.1677	–0.51	<0.000001*
Nitrate (μM)	–0.43	<0.000001*	–0.16	0.0512
Phosphate (μM)	–0.44	<0.000001*	–0.64	<0.000001*
Silicate (μM)	0.076	0.4005	–0.067	0.4626
Ammonium (μM)	–0.18	0.0443	0.069	0.4458
Total N (μM)	–0.087	0.3357	0.27	0.0028
DOC (μM)	0.20	0.0398	0.62	<0.000001*
Chl *a* (μg l^-1^)	0.42	<0.000001*	0.35	0.0001*
Phytoplankton biomass (mgC m^-3^)	0.40	<0.000001*	0.15	0.1038
Bacterial abundance (cells ml^-1^)	0.28	0.0018	na	na
Bacterial heterotrophic production (μg C l^-1^ h^-1^)	na	na	0.28	0.0018
Diatoms (mgC m^-3^)	–0.18	0.0468	–0.19	0.0376
Dinoflagellates (mgC m^-3^)	0.034	0.7084	–0.20	0.0284
Cyanobacteria (mgC m^-3^)	0.66	<0.000001*	0.43	<0.000001*
Golden algae (mgC m^-3^)	–0.23	0.0103	0.027	0.7696
Flagellates (mgC m^-3^)	0.60	<0.000001*	0.49	<0.000001*

*Asterisks indicate highly robust correlations (*ρ* > 0.25, adjusted *p*-values after Bonferroni correction *p*^∗^ < 0.05). Further details are shown in Supplementary Figures 2, 3. Note that total N and DOC data is comprised of slightly fewer samplings.*

### Nutrient Limitation

**FIGURE 4:**
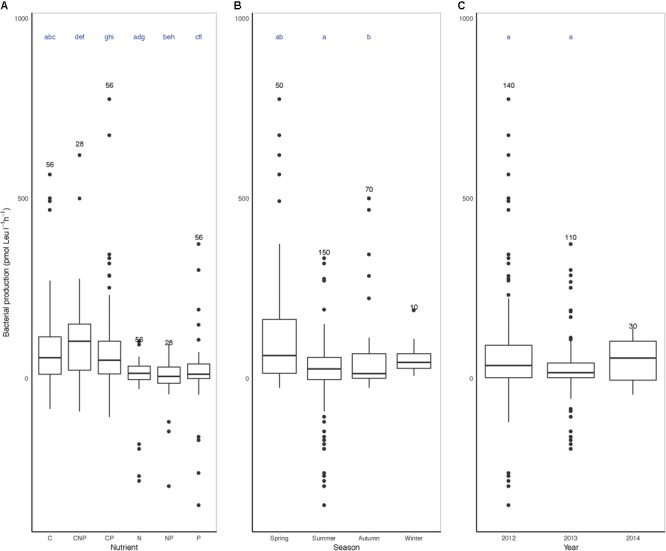
Boxplots of nutrient limitation bioassays grouped by season, year, or nutrient. Nutrient limitation assays, bacterial heterotrophic production (pmol Leu l^-1^ h^-1^) corrected for control incubations after 24 h incubation with added nutrients grouped by **(A)** nutrient, **(B)** season, and **(C)** year. In the nutrient limitation assays, C denotes carbon, P denotes phosphor, and N denotes nitrogen, or a combination of the above. The number of measurements per group is indicated above the boxplot and includes biological replicates as separate data points (#*n*). Letters above the boxplots indicate significant differences between the groups sharing a letter (i.e., seasons, years, or nutrients) (Monte Carlo simulation randomization test considered significantly different when ^∗^*p* < 0.05).

In the nutrient limitation bioassays, we considered a response in bacterial heterotrophic production to nutrient additions in comparison to control incubations as a proxy for growth limitation. Monte Carlo simulation randomization test resulted in a significant trend for nutrient limitation during spring, as compared to summer and autumn and differences in 2012 as compared to 2013 (Figure 4 and Supplementary Figure 5). The major limiting nutrient was carbon (C, CNP, and CP treatments) as compared to nitrogen or phosphorus (N, NP, and P treatments) (Figure 4). In fact, particularly N seemed to be the least limiting nutrient for heterotrophic biomass production. N and P additions exclusively did not have a positive effect on bacterial heterotrophic production until mid-June 2012 (Supplementary Figure 5). In April 2013, C, N, and P were all limiting bacterial heterotrophic production. From June to September 2013, C was not exclusively limiting the heterotrophic community, although N and P frequently limited bacterial heterotrophic production. In Autumn 2013 and summer 2014, responses in the nutrient limitation assays were less explicit.

### Organic Substrate Hydrolysis and Uptake

**FIGURE 5:**
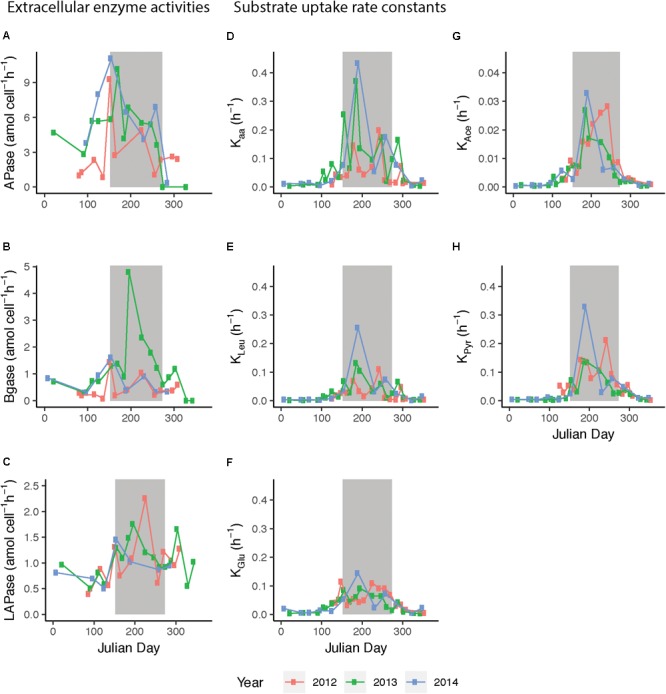
Extracellular enzymatic activities and substrate uptake rate constants during 2012–2014, plotted against day of year (Julian day). Enzyme activities/uptake rate constants of **(A)** alkaline phosphatase (APase), **(B)** β-glucosidase (BGase), **(C)** leucine aminopeptidase (LApase), **(D)** amino acids (*K*_aa_), **(E)** leucine (*K*_Leu_), **(F)** glucose (*K*_Glu_), **(G)** acetate (*K*_Ace_), and **(H)** pyruvate (*K*_Pyr_). 2012 data are shown in red, 2013 data in green, and 2014 data in blue. Bulk extracellular enzyme activity data from March 2012 to August 2013 were previously published in Baltar et al. (2016). Presented are average values of three technical replicates, gray background indicates summer months.

Enzyme activities were significantly different between seasons and between the enzymes (Monte Carlo simulation randomization test, Supplementary Figure 6). APase activity peaked in early summer (up to 11.1 amol cell^-1^ h^-1^ in June 2014) while during late summer the activity remained below 8.0 amol cell^-1^ h^-1^ (Figure 5A). Beta-glucosidase (BGase) was highest during summer 2013 (up to 4.8 amol cell^-1^ h^-1^) and also showed initial peaks in early June (Figure 5B). Leucine aminopeptidase (LAPase) activity increased in early June, and thereafter it stayed above winter and spring values until autumn (LAPase peaked at 2.26 amol cell^-1^ h^-1^ in August 2012) (Figure 5C).

Uptake rate constants (*K*) of the five monomeric substrates studied were elevated during summer and varied significantly between seasons and substrates (Monte Carlo simulation randomization test, Supplementary Figure 6). Moreover, rate constants varied notably over short time spans during the productive season and the patterns appeared different between years (Figure 5). During summer, the uptake rate constants for both glucose (*K*_Glu_) and leucine (*K*_Leu_) ranged from 0.05 to 0.1 h^-1^, with intermittent peaks up to 0.15 and 0.25 h^-1^. The *K*_Leu_ and the *K* for amino acids (*K*_aa_) slowly increased upon spring blooms in April until they reached an early summer peak. Interestingly, the *K* for pyruvate (*K*_Pyr_) was often higher than for *K*_Leu_ and *K*_Glu_ during summer. The amplitude in variability in acetate was lower than for the other substrates and the values were lower compared to the other *K* values (maximum in July ∼0.03 h^-1^). Intriguingly, in both 2012 and 2013, temperature drops in September were mirrored by decreased *K*-values.

Uptake rate constants of all substrates correlated significantly with bacterial heterotrophic production, cyanobacteria biomass, temperature, and phosphate (Spearman’s rank correlation test, Supplementary Figure 7). In contrast, uptake rate constants were not significantly correlated to DOC, Chl *a*, or bacterial biomass (Supplementary Figures 7, 8). Among the substrates, *K*_Leu_ showed the strongest correlation to *K*_aa_, and similarly, *K*_Ace_ and *K*_Pyr_ were strongly correlated to each other (Supplementary Figure 7). Furthermore, glucose correlated more strongly to *K*_Ace_ and *K*_Pyr_ compared to *K*_Leu_ or *K*_aa_ (Supplementary Figure 7).

## Discussion

In this multi-year study, the high-frequency sampling schedule allowed uncovering a number of unexpected features in biological variables, emphasizing the extraordinary ecological responsiveness of microbial succession to environmental drivers. Accordingly we note: (i) pronounced variation in species dominance and amplitude of phytoplankton blooms, (ii) marked differences in dynamics of bacterial production and abundance following spring compared to summer phytoplankton blooms, with notably persistent periods of increases/decreases, (iii) noticeable influence of elevated phosphate input during winter on the microbial community, and (iv) high variability over short time scales in organic substrate hydrolysis and uptake. These observations formed an important complement to, and an ample extension of, the seasonally recurrent patterns in physicochemical and biological variables typically observed in temperate waters, in the Baltic Proper surface waters, and elsewhere [see for example Tiselius et al. (2015)]. These recurrent patterns included the seasonal dynamics in inorganic nutrient concentrations and phytoplankton blooms (Tiselius et al., 2015; Teeling et al., 2016; Bunse and Pinhassi, 2017).

### Variation in Species Dominance and Amplitude of Phytoplankton Blooms

In our high-frequency study, the timing of the spring and summer blooms was highly consistent between years, irrespectively of phytoplankton community composition. Notably, the variability in Chl *a* concentrations during the spring bloom month April was fourfold higher than during the summer bloom month July (reflected also, but to a lesser extent, in phytoplankton biomass). Moreover, phytoplankton community composition in April was more variable (due to shifts in dominance of diatoms and dinoflagellates between years) as compared to July (consistent dominance of filamentous cyanobacteria). This agrees with recent reports that the Baltic Sea spring bloom has been shifting significantly in community structure and intensity over the last decades, and that the spring dominance of diatoms as compared to dinoflagellates appears to change with approximately decadal oscillations (Suikkanen et al., 2007; Wasmund et al., 2011). Also in the Western Baltic Proper, warmer winters have recently resulted in a trend toward higher dinoflagellate and less diatom biomass during spring (Wasmund et al., 2011; Legrand et al., 2015), potentially superimposed on more long-term oscillations. Changes in the phytoplankton community composition is likely to influence both ecological interactions in the pelagic food web (e.g., changing the conditions for competitors and/or grazers) and the amount of organic material that is sinking out of the photic zone (Tamelander and Heiskanen, 2004). We suggest that the high variability in the ecology of the phytoplankton spring blooms (especially with respect to amplitude and composition of blooms) compared to the summer bloom dynamics can influence how general carbon fluxes or specific microbial responses are differentially regulated across productive seasons.

During summer, nitrogen fixation by filamentous cyanobacteria is a main source of nitrogen to the Baltic Sea aquatic system, acting to increase otherwise low N:P ratios (Vahtera et al., 2007; Karlson et al., 2015). In this study, filamentous cyanobacteria dominated the summer phytoplankton biomass. While the Chl *a* values during June–September were slightly lower as reported for the Western Gotland Basin, trends of higher Chl *a* variance in 2011 compared to 2012 and 2013 were in agreement with the HELCOM monitoring report (HELCOM, 2018). The somewhat lower concentrations reported in our study might depend on the different sampling methodologies rather than solely indicating differing sampling frequencies; namely Chl *a* measured from discrete 2 m depth in our study compared to the combined estimate of *in situ* sampling of 0–10 m surface water, FerryBox data, and Earth Observation (EO) remote sensing satellite data as conducted by HELCOM (2018). Yet, the high-frequency sampling reported in this study at one sole station allowed distinguishing two cyanobacterial biomass peaks; the first peak typically appeared in early July and the second peak observed in the end of July/beginning of August. Shifts in cyanobacterial community composition during the course of the Baltic Sea summer were recently reported; in the study of Bertos-Fortis et al. (2016), several heterocystous taxa co-occurred in July, with mainly *Aphanizomenon* sp. dominating later in August (Bertos-Fortis et al., 2016). These twin peaks in biomass and a succession of cyanobacteria species could provide different ecosystem functions (such as nitrogen fixation rates, release of different organic carbon compounds, or toxic substances) that might further affect heterotrophic bacterioplankton community functioning and composition.

Protists like pikoeukaryotes and nanoflagellates are important components of the marine food web, and modern molecular analyses are providing exciting insight into their diversity and dynamics (Worden and Not, 2008; Not et al., 2009; Berdjeb et al., 2018; Yang et al., 2018). Previous studies in different regions in the Baltic Sea have reported long-term increases in nanoflagellates from 1972 to 2003 (Suikkanen et al., 2007), but few studies of the Baltic Sea explicitly discuss the seasonal dynamics in this microbial compartment (Kuosa, 1991; Piwosz and Pernthaler, 2010). In our study, the extent of nanoflagellate dominance of the phytoplankton biomass in late summer and autumn was highly surprising – indicating this plankton fraction might be overlooked with regards to its ecological role for structuring natural bacterioplankton communities.

The relative proportion of flagellates contributed well above 80% to autumn phytoplankton biomass during an extended period, even though the overall phytoplankton biomass was low after summer. Samuelsson et al. (2006) showed that heterotrophic flagellate biomass increased with higher primary production in the northern Baltic Proper, and that the growth of flagellates was limited by bacterial biomass and temperature (Samuelsson et al., 2006). Thus, if major portions of the flagellates were heterotrophic or mixotrophic, it is possible that the increase in relative abundance of flagellates we observed from late summer is linked with elevated temperatures and bacterial growth at this time. In a reciprocal manner, flagellate grazing would then influence the temporal dynamics in bacterioplankton biomass and production. Another consequence of the high relative abundance of flagellates is that it could contribute to explain that Chl *a* concentrations were more variable compared to phytoplankton biomass in autumn. Regardless whether the observed flagellates were autotrophic (carrying out photosynthesis) and potentially producing DOM, heterotrophic and grazing on bacteria, or mixotrophic, their abundance and heterogeneity could have important effects on bacterioplankton activities during this time of the year. This issue represents an important venue for the future.

### Bacterial Heterotrophic Production and Bacterial Abundance Over the Seasons

We observed pronounced seasonal dynamics in both bacterial heterotrophic production and abundance during the study period. Before the spring bloom, bacteria did not show considerable net growth, underlining the bacterioplankton dependence on phytoplankton and consecutively autochthonous DOM in the Western Baltic Proper. Following the phytoplankton spring bloom peak, bacterial heterotrophic production peaked after a 0–14 days delay whereas in summer only 0–4 days delay to the phytoplankton bloom was observed. Such quicker response in summer could be due to higher temperatures but could also reflect differences in DOM quantity/quality or different abilities to respond by different taxonomic groups of bacteria growing in summer compared to spring. Interestingly, during the twin cyanobacteria peaks in summer, bacterial heterotrophic production increased with the first peak (mid-July), while bacterioplankton biomass increased with the second peak (August). This imparity could result from compositional changes in the cyanobacterial community over time; providing bacteria with different DOM qualities or by producing different antimicrobial substrates [e.g., *Nodularia* and *Dolichospermum* species in the Baltic Proper produce different potent toxins (Fewer et al., 2009; Mazur-Marzec et al., 2015)].

Effects of phage infection or flagellate grazing could also play in to regulate the observed pronounced differences in bacterial growth dynamics in relation to different phytoplankton blooms. Bacterial biomass showed remarkable variability over the productive seasons – although there was a general increase from April until the end of August/September. Still, there were recurrent periods of lower bacterial abundances. Seemingly, the overall bacterioplankton biomass was additively supported by several events over the productive season, such as the spring bloom, subsequent temperature stimulation of bacterial heterotrophic production, and again after the summer phytoplankton blooms. Few studies have investigated seasonal dynamics in bacterial heterotrophic production or abundance/biomass in surface waters of the Baltic Proper, yet there are reports from various other basins, like the Bothnian Bay, Bothnian Sea, and German Bight (Kuosa and Kivi, 1989; Pinhassi and Hagström, 2000; Algesten et al., 2004; Wikner and Andersson, 2012; Hoppe et al., 2013; Huseby and Wikner, 2015). In the Kiel Bight, bacterial abundances ranged between 0.5 to 6 × 10^6^ cells ml^-1^ over 20 years and exhibited highest values during summer (Hoppe et al., 2013), which is similar to the abundances observed in our study. Higher bacterial abundances and production values during summer compared to winter, and positive correlation between production and temperature were previously found in the northern Bothnian Sea; but as found in the Bothnian Bay, highest production rates can also take place in spring (Zweifel et al., 1993; Pinhassi and Hagström, 2000; Algesten et al., 2004).

Actually, it has been suggested that DOC accumulates in spring both due to phytoplankton-derived (autochthonous) DOC production and due to river discharge of allochthonous DOC (Zweifel et al., 1995; Seidel et al., 2017). Still, bacteria accomplished net removal of accumulated DOC during summer (Zweifel et al., 1993, 1995), demonstrating the critical role of bacteria in the Baltic Sea carbon cycle. Especially in the northern basins with high riverine DOC input (e.g., Gulf of Bothnia), it has been postulated that the pelagic system is net heterotrophic (Algesten et al., 2004; Wikner and Andersson, 2012; Seidel et al., 2017). Further, upon increasing river-water discharges including humic substances in the Bothnian Bay, a decrease in phytoplankton production has been found to coincide with increased yearly bacterioplankton to phytoplankton biomass production ratios (Wikner and Andersson, 2012). This study also revealed that DOC concentrations increased from spring to summer and our results further agree with the general notion that bacteria account for higher portions of microbial biomass when total primary productivity is lower (Gasol et al., 1997; Church, 2008). Moreover, our findings of pronounced dynamics in both bacterial abundance and production on the level of weeks emphasize that the Baltic Sea Proper bacteria are continuously encountering changes in growth conditions. Thus, while, e.g., monthly or annual biomass averages serve an important purpose in many contexts (e.g., models), such averages by necessity ignore short-term variability. We pose that understanding microbial dynamics and their ecological drivers influencing community productivities over short time scales is important as they may cumulatively influence biogeochemical processes.

### Influence of Elevated Phosphate Input During Winter

Inorganic nutrient concentrations in temperate waters typically vary on a yearly basis – yet, the phosphate concentrations showed a noteworthy increase in December 2011 as compared to the other years. These elevated surface water concentrations of phosphate potentially resulted from unusually strong storms in the end of November and in December 2011, causing extensive wave height anomalies (Pettersson et al., 2012; Siegel and Gerth, 2012; Bobykina and Stont, 2015) and likely vertical mixing of the water column. We interpret the low APase activity between mid-March and mid-May of 2012, compared to the same time period in other years, as a response to this elevated phosphate concentration. Moreover, the nutrient limitation bioassay experiments indicated that the bacterioplankton was not limited by P or N during the spring of 2012, but rather was limited by the access to labile organic carbon. Incidentally, bacterial heterotrophic production was higher during April–May 2012 compared to the other years, potentially indicating a relatively weak bottom-up control at this time.

It is generally recognized that the availability of (in)organic nutrients has decisive influence on bacterioplankton growth and community composition (Church et al., 2000; Pinhassi et al., 2006; Allen et al., 2012; Karl, 2014). While small amounts of P addition in the P-limited Mediterranean Sea affect the bacterial community composition only minimally over short times (Fodelianakis et al., 2014), excess phosphate loading or even reactive phosphate concentrations above 0.4 μM can enhance the speed of temporal succession of bacterioplankton and stimulate growth of bacteria strains with high RNA content, respectively (Chen et al., 2016; Liu and Liu, 2016). Whereas such high phosphate concentrations were measured frequently during winter at LMO, we did not detect correlatingly elevated bacterioplankton productivities (Supplementary Figure 9), likely due to temperature limitations in winter that inhibited overall bacterial activities and growth. In fact, the proportion of dissolved extracellular enzyme activities as compared to bulk extracellular enzyme activities is elevated during winter as compared to summer seasons and correlates negatively to temperature, suggesting a decoupling of hydrolysis rates from microbial dynamics during winter (Baltar et al., 2016). Of particular importance in the Baltic Sea is that increased access to P in summer stimulates growth of filamentous cyanobacteria (Granéli et al., 1990; Andersson et al., 2015), which can lead to pronounced cyanobacteria blooms. We infer that experimental exploration of the linkages between bacterioplankton nutrient limitation and the nutrient stimulation of phytoplankton growth could provide important insights into the regulation of microbial bloom dynamics.

### Organic Substrate Hydrolysis and Uptake

Generally, different phytoplankton groups produce different DOM suites which in turn select for different marine bacterioplankton populations and communities (Pinhassi et al., 2004; Sarmento and Gasol, 2012; Teeling et al., 2012; Becker et al., 2014; Xing et al., 2015). Temporal differences in substrate uptake efficiencies between glucose and leucine uptake rates have for example been observed following spring blooms (Alonso and Pernthaler, 2006a,b). Bacterial taxa selection is thus likely, in part, governed by factors such as polymer degradation capacity, utilization of carbon compound monomers, as well as varying chemotactic abilities toward organic matter hotspots among bacteria (Pinhassi et al., 1999; Fandino et al., 2001; Alderkamp et al., 2007; Teeling et al., 2012; Smriga et al., 2016). Further, phylogenetic groups of bacteria markedly differ in their uptake rate activities throughout the year (Alonso-Sáez and Gasol, 2007) and microcosm experiments in the Mediterranean and the Baltic Sea showed that these carbon compounds stimulated the growth of different specialist and generalist bacteria, suggesting distinctive ecological roles of different bacterial taxa in the turnover of DOM (Gómez-Consarnau et al., 2012).

If above principles apply in our study, the different substrates could be consumed by distinct bacterial groups, or different carboxylic acids might be taken up by analogous transport mechanisms as compared to saccharides or amino acids. In fact, our current analysis showed that the bulk bacterial uptake rate constants for multiple sets of studied carbon compounds over 3 years differed dynamically between substrates and changed seasonally. These results substantially expand the findings from previous studies that have on one hand investigated in important detail the uptake rates and concentrations of single carbon compounds, but on the other hand have been limited in time or the number of compounds/compound classes analyzed in parallel [see for example Alonso and Pernthaler (2006b) and Vila-Costa et al. (2006)]. The carbon compounds studied here (glucose, the carboxylic acids acetate and pyruvate, and amino acids), each account for up to 15% of the daily uptake of organic carbon by bacteria in various sea areas (Suttle et al., 1991; Rich et al., 1996; Obernosterer et al., 1999; Skoog et al., 1999; Ho et al., 2002; Simó et al., 2002), indicating that they are ecologically important to marine bacteria. Different bacterial groups have been shown to have distinct and dynamic DOM uptake patterns, especially during nutrient-limiting summer periods, that were further hypothesized to result from different affinity uptake systems or microdiversity patterns within bacterial groups (Alonso-Sáez and Gasol, 2007). Here, we elucidated that the uptake rate constants were generally higher during summer when cyanobacteria bloomed and temperatures were elevated as compared to the spring bloom period also in the Baltic Sea.

Curiously, glucose and carboxylic acids displayed stronger correlations to bacterial heterotrophic production than leucine and amino acids, pointing to differences in methodology between substrate uptake rates (where leucine was provided in trace concentrations) and bacterial heterotrophic production estimates (where leucine was provided at saturating levels). Thus, the bacterial heterotrophic production is a measure of the community biomass production, while the differences between substrate uptake rate constant of the different substrates can be related to functional traits of the bacterial community. Lastly, environmental or microbial parameters such as Chl *a* or bacterial abundances did not robustly explain the variability in substrate preference or uptake rate constants in our dataset. This may appear surprising given the dependence of bacteria on phytoplankton DOM release, but could reflect a highly dynamic microbial community consisting of various heterotrophic bacteria, far more specific in their heterotrophic nature than previously anticipated.

Reports from high resolution time series recently revealed that gene expression patterns of marine microbial communities can change within hours and on diurnal cycles, while changes in bacterial community composition and dynamics may vary over days to weeks (Mayali et al., 2010; Ottesen et al., 2011, 2014; Needham et al., 2013; Lindh et al., 2015; Needham and Fuhrman, 2016; Martin-Platero et al., 2018). On the other hand, marine bacterioplankton communities show seasonal succession patterns and to a great extend recur annually (Fuhrman et al., 2006; Hewson et al., 2006; Gilbert et al., 2012; Fuhrman et al., 2015; Ward et al., 2017). Thus, the vast short-term variability in our dataset and of the measured microbial productivities and cell abundance estimates likely is the result of the combined influence of gene expression and community composition changes. Reciprocally this means that cumulative changes in functional genes and proteins (expression and abundances due to community turnover) would affect net productivities and stocks of plankton masses at large scales. Moreover, complex biological food-web interactions, such as grazing by bacterivorous flagellates or infecting phages will further influence bacterioplankton dynamics. Addressing taxonomic and functional diversities in relation to substrate utilization rates will provide further insights into the spatio-temporal variability of microbial systems and its ecosystem functions.

## Conclusion

Environmental surface water conditions change on semidiurnal, daily, and seasonal scales (Gunderson et al., 2016). Traditional time series based on monthly sampling have established the importance and persistence of seasonal fluctuations in physical, chemical, and biological parameters in several temperate marine areas (Fuhrman et al., 2015; Bunse and Pinhassi, 2017). Further, marine microbial times-series studies have recently shown the importance of high-frequency sampling to disentangle changes in bacterioplankton communities and related gene expression patterns (Teeling et al., 2012, 2016; Aylward et al., 2015; Lindh et al., 2015; Needham and Fuhrman, 2016; Ward et al., 2017; Martin-Platero et al., 2018). In this high-resolution time series in the Baltic Sea, we showed that monthly sampling frequencies are adequate to capture the variability in a number of physical or chemical variables in surface seawater across years, such as temperature. Yet, it appears that there is extensive biological variability in microbial parameters, often occurring on time scales of days to a few weeks, that would be missed by sampling at monthly intervals. This could lead to over-or underestimation of microbial abundances, productivities, or ecological functions.

Bacterial growth was influenced by nutrient availability, temperature, as well as several successive phytoplankton bloom events leading to pronounced bacterial abundance increases. Reciprocally, bacteria feasibly influenced nutrients and organic substrate stocks and potentially phytoplankton communities. Curiously, during summer stratification with stable physicochemical surface water conditions, we observed the highest heterotrophic rates (such as organic substrate hydrolysis and uptake) but also the highest variability in biological parameters. This indicates that the dependence of biology on physicochemical conditions is complex and may be driven to important portions by ecological interactions in the microbial food web, and even with potential linkages to higher trophic levels. Ultimately, knowledge of the complexity of bacterial community functioning responses to physicochemical and biological factors associated with seasonality will facilitate the understanding of microbial processes and its implications for fluxes of energy and matter in marine food webs. Intriguingly, sampling and data collection at these frequencies may also facilitate the development of biogeochemical- and food web models incorporating bacterioplankton dynamics. This would allow for a better understanding and prediction of ecosystem-wide responses to environmental disturbances in the Baltic Sea.

## Author Contributions

ML, CL, and JP conceived the time-series study. SI, CB, FB, EF, ML, and SM-G processed samples in laboratory. SI, CB, FB, MB-F, EF, SM-G, ML, EL, CL, and JP analyzed and interpreted the data. CB, SI, and JP wrote the manuscript with the help of the other co-authors. All authors have read and significantly contributed to the manuscript.

## Conflict of Interest Statement

The authors declare that the research was conducted in the absence of any commercial or financial relationships that could be construed as a potential conflict of interest.

## Supplementary Material

FIGURE S1 Linegraph of **(A)** phosphate and **(B)** APase activity over time.

FIGURE S2 Spearman’s rank correlation test results of abiotic and biotic variables against each other 2011–2014, excluding the variable DOC. Spearman’s *rho* is indicated as “Cor,” *p*-values are indicated as “p.” Colors denote significance thresholds: red *p*-values denote significant values after Bonferroni correction ^∗^*p* > 0.05, red Cor denote Spearman’s *rho* values > 0.45, blue denotes Spearman’s *rho* values > 0.25.

FIGURE S3 Spearman’s rank correlation test results of abiotic and biotic variables against each other including DOC values but excluding 2011 data. Spearman’s *rho* is indicated as “Cor,” *p*-values are indicated as “p.” Colors denote significance thresholds: red *p*-values denote significant values after Bonferroni correction ^∗^*p* > 0.05, red Cor denote Spearman’s *rho* values > 0.45, blue denotes Spearman’s *rho* values > 0.25.

FIGURE S4 Subsampled data during 2011–2014. All data points that were sampled during the first week of the month (days 1–7) are labeled in red, underlying black points indicate all measured data for temperature (°C), bacterial heterotrophic production (μg C l^-1^ h^-1^), Chl *a* (μg l^-1^), and phytoplankton biomass (mgC m^-3^).

FIGURE S5 Nutrient limitation assays during 2012–2014. Displayed are the bacterial heterotrophic production estimates (pmol Leu l^-1^ h^-1^) of single or combined nutrient addition assays after 24 h incubation (biological replicates 1 or 2, respectively): N treatments (ammonium), P treatments (phosphate), and C treatments (glucose). Error bars indicate standard deviation of technical triplicates.

FIGURE S6 Boxplots of enzyme activities and substrate uptake rate constants, grouped by season, year, or enzyme/substrate. Enzyme activities normalized to bacterial abundances (amol cell^-1^ h^-1^) grouped by **(A)** enzyme, **(C)** season, and **(E)** year. APase denotes alkaline phosphatase, BGase denotes β-glucosidase, and LApase denotes leucine aminopeptidase. Substrate uptake rate constants *K* (h^-1^) grouped by **(B)** substrate, **(D)** season, and **(F)** year; acetate (*K*_Ace_), amino acids (*K*_aa_), glucose (*K*_Glu_), leucine (*K*_Leu_), and pyruvate (*K*_Pyr_). The number of measurements per group is indicated above the boxplot and includes biological replicates as separate data points (#*n*). Letters above the boxplots indicate significant differences between seasons, years, or substrates/nutrients/enzymes (Monte Carlo simulation randomization test considered significantly different when ^∗^*p* < 0.05).

FIGURE S7 Spearman’s rank correlation test results of substrate uptake rate constants and biologically relevant variables excluding DOC. Spearman’s *rho* is indicated as “Cor,” *p*-values are indicated as “p.” Colors denote significance thresholds: red *p*-values denote significant values after Bonferroni correction ^∗^*p* > 0.05, red Cor denote Spearman’s *rho* values > 0.45, blue denotes Spearman’s *rho* values > 0.25.

FIGURE S8 Spearman’s rank correlation test results of substrate uptake rate constants and biologically relevant variables including DOC. Spearman’s *rho* is indicated as “Cor”, p-values are indicated as “p”. Colors denote significance thresholds: red *p*-values denote significant values after Bonferroni correction ^∗^*p* > 0.05, red Cor denote Spearman’s *rho* values > 0.45, blue denotes Spearman’s *rho* values > 0.25.

FIGURE S9 Graph of bacterial productivities and abundances plotted against phosphate concentrations during 2011–2014. **(A)** APase, **(B)** bacterial production, and **(C)** cell abundances. The color gradients denote temperatures at time of sampling.

TABLE S1 Chl a concentrations during summer time at LMO. Samples derive from 2m water depth and during the time between June and September.

## References

[B1] Alderkamp A. C. Van Rijssel M. Bolhuis H. (2007). Characterization of marine bacteria and the activity of their enzyme systems involved in degradation of the algal storage glucan laminarin. *FEMS Microbiol. Ecol.* 59 108–117. 10.1111/j.1574-6941.2006.00219.x 17233748

[B2] Algesten G. Wikner J. Sobek S. Tranvik L. J. Jansson M. (2004). Seasonal variation of CO2 saturation in the Gulf of Bothnia: indications of marine net heterotrophy. *Glob. Biogeochem. Cycles* 18 4021–4028. 10.1029/2004GB002232

[B3] Allen L. Z. Allen E. E. Badger J. H. McCrow J. P. Paulsen I. T. Elbourne L. D. (2012). Influence of nutrients and currents on the genomic composition of microbes across an upwelling mosaic. *ISME J.* 6 1403–1414. 10.1038/ismej.2011.201 22278668 PMC3379637

[B4] Alneberg J. Karlsson C. Divne A.-M. Bergin C. Homa F. Lindh M. (2018a). Genomes from uncultivated prokaryotes: a comparison of metagenome-assembled and single-amplified genomes. *Microbiome* 6:173. 10.1186/s40168-018-0550-0 30266101 PMC6162917

[B5] Alneberg J. Sundh J. Bennke C. Beier S. Lundin D. Hugerth L. (2018b). BARM and BalticMicrobeDB, a reference metagenome and interface to meta-omic data for the Baltic Sea. *Sci. Data* 5:18014. 10.1038/sdata.2018.146 30063227 PMC6067050

[B6] Alonso C. Pernthaler J. (2006a). Concentration-dependent patterns of leucine incorporation by coastal picoplankton. *Appl. Environ. Microbiol.* 72 2141–2147. 16517664 10.1128/AEM.72.3.2141-2147.2006 PMC1393217

[B7] Alonso C. Pernthaler J. (2006b). Roseobacter and SAR11 dominate microbial glucose uptake in coastal North Sea waters. *Environ. Microbiol.* 8 2022–2030. 17014500 10.1111/j.1462-2920.2006.01082.x

[B8] Alonso-Sáez L. Gasol J. M. (2007). Seasonal variations in the contributions of different bacterial groups to the uptake of low-molecular-weight compounds in northwestern Mediterranean coastal waters. *Appl. Environ. Microbiol.* 73 3528–3535. 10.1128/AEM.02627-06 17400772 PMC1932672

[B9] Andersson A. Höglander H. Karlsson C. Huseby S. (2015). Key role of phosphorus and nitrogen in regulating cyanobacterial community composition in the northern Baltic Sea. *Estuar. Coast. Mar. Sci.* 164 161–171. 10.1016/j.ecss.2015.07.013

[B10] Aylward F. O. Eppley J. M. Smith J. M. Chavez F. P. Scholin C. A. DeLong E. F. (2015). Microbial community transcriptional networks are conserved in three domains at ocean basin scales. *Proc. Natl. Acad. Sci. U.S.A.* 112 5446–5448. 10.1073/pnas.1502883112 25775583 PMC4418921

[B11] Azam F. (1998). Microbial control of oceanic carbon flux: the plot thickens. *Science* 280 694–696. 10.1126/science.280.5364.694

[B12] Azam F. Fenchel T. Field J. G. Gray J. S. Meyer-Reil L. A. Thingstad F. (1983). The ecological role of water-column microbes in the sea. *Mar. Ecol. Prog. Ser.* 10 257–263. 10.3354/meps010257

[B13] Backer H. Leppänen J.-M. Brusendorff A. C. Forsius K. Stankiewicz M. Mehtonen J. (2010). HELCOM Baltic Sea action plan–A regional programme of measures for the marine environment based on the ecosystem approach. *Mar. Pollut. Bull.* 60 642–649. 10.1016/j.marpolbul.2009.11.016 20006361

[B14] Baines S. B. Pace M. L. (1991). The production of dissolved organic matter by phytoplankton and its importance to bacteria: patterns across marine and freshwater systems. *Limnol. Oceanogr.* 36 1078–1090. 10.4319/lo.1991.36.6.1078

[B15] Baltar F. Legrand C. Pinhassi J. (2016). Cell-free extracellular enzymatic activity is linked to seasonal temperature changes: a case study in the Baltic Sea. *Biogeosciences* 13 2815–2821. 10.5194/bg-13-2815-2016

[B16] Becker J. W. Berube P. M. Follett C. L. Waterbury J. B. Chisholm S. W. DeLong E. F. (2014). Closely related phytoplankton species produce similar suites of dissolved organic matter. *Front. Microbiol.* 5:111. 10.3389/fmicb.2014.00111 24748874 PMC3975126

[B17] Behrenfeld M. J. Boss E. Siegel D. A. Shea D. M. (2005). Carbon-based ocean productivity and phytoplankton physiology from space. *Glob. Biogeochem. Cycles* 19:GB1006 10.3389/fmicb.2017.01926

[B18] Berdjeb L. Parada A. Needham D. M. Fuhrman J. A. (2018). Short-term dynamics and interactions of marine protist communities during the spring–summer transition. *ISME J.* 12 1907–1917. 10.1038/s41396-018-0097-x 29599520 PMC6052004

[B19] Bertos-Fortis M. Farnelid H. M. Lindh M. V. Casini M. Andersson A. Pinhassi J. (2016). Unscrambling cyanobacteria community dynamics related to environmental factors. *Front. Microbiol.* 7:625. 10.3389/fmicb.2016.00625 27242679 PMC4860504

[B20] Bianchi T. S. Engelhaupt E. Westman P. Andren T. Rolff C. (2000). Cyanobacterial blooms in the Baltic Sea: natiral or human-induced? *Limnol. Oceanogr.* 45 716–726. 10.4319/lo.2000.45.3.0716

[B21] Bobykina V. P. Stont Z. I. (2015). Winter storm activity in 2011–2012 and its consequences for the Southeastern Baltic coast. *Water Res.* 42 371–377. 10.1134/s0097807815030021

[B22] Bunse C. Pinhassi J. (2017). Marine bacterioplankton seasonal succession dynamics. *Trends Microbiol.* 25 494–505. 10.1016/j.tim.2016.12.013 28108182

[B23] Celepli N. Sundh J. Ekman M. Dupont C. L. Yooseph S. Bergman B. (2017). Meta-omic analyses of Baltic Sea cyanobacteria: diversity, community structure and salt acclimation. *Environ. Microbiol.* 19 673–686. 10.1111/1462-2920.13592 27871145

[B24] Chen X. Wang K. Guo A. Dong Z. Zhao Q. Zhang D. (2016). Excess phosphate loading shifts bacterioplankton community composition in oligotrophic coastal water microcosms over time. *J. Exp. Mar. Biol. Ecol.* 483 139–146. 10.1016/j.jembe.2016.07.009

[B25] Church M. J. (2008). “Resource control of bacterial dynamics in the sea,” in *Microbial Ecology of the Oceans* 2nd Edn ed. Kirchman D. L. (Hoboken, NJ: Wiley-Liss) 335–382. 10.1002/9780470281840.ch10

[B26] Church M. J. Hutchins D. A. Ducklow H. W. (2000). Limitation of bacterial growth by dissolved organic matter and iron in the Southern Ocean. *Appl. Environ. Microbiol.* 66 455–466. 10.1128/AEM.66.2.455-466.2000 10653704 PMC91849

[B27] Cole J. J. Findlay S. Pace M. L. (1988). Bacterial production in fresh and saltwater ecosystems: a cross-system overview. *Mar. Ecol. Prog. Ser.* 43 1–10. 10.3354/meps043001

[B28] Edler L. (ed.) (1979). “Recommendations on methods for marine biological studies in the Baltic Sea,” in *Phytoplankton and Chlorophyll* (Sweden: Baltic Marine Biologists BMB) 1–38.

[B29] Fagerbakke K. Heldal M. Norland S. (1996). Content of carbon, nitrogen, oxygen, sulfur and phosphorous in native and cultured bacteria. *Aquat. Microb. Ecol.* 10 15–27. 10.3354/ame010015

[B30] Fandino L. B. Riemann L. Steward G. F. Long R. A. Azam F. (2001). Variations in bacterial community structure during a dinoflagellate bloom analyzed by DGGE and 16S rDNA sequencing. *Aquat. Microb. Ecol.* 23:119 10.3354/ame023119

[B31] Fewer D. P. Köykkä M. Halinen K. Jokela J. Lyra C. Sivonen K. (2009). Culture-independent evidence for the persistent presence and genetic diversity of microcystin-producing *Anabaena* (Cyanobacteria) in the Gulf of Finland. *Environ. Microbiol.* 11 855–866. 10.1111/j.1462-2920.2008.01806.x 19128321

[B32] Fodelianakis S. Pitta P. Thingstad T. Kasapidis P. Karakassis I. Ladoukakis E. (2014). Phosphate addition has minimal short-term effects on bacterioplankton community structure of the P-starved Eastern Mediterranean. *Aquat. Microb. Ecol.* 72 98–104. 10.3354/ame01693

[B33] Fox J. Weisberg S. (2011). *An {R} Companion to Applied Regression.* Thousand Oaks CA: Sage.

[B34] Fuhrman J. A. Cram J. A. Needham D. M. (2015). Marine microbial community dynamics and their ecological interpretation. *Nat. Rev. Microbiol.* 13 133–146. 10.1038/nrmicro3417 25659323

[B35] Fuhrman J. A. Hewson I. Schwalbach M. S. Steele J. A. Brown M. V. Naeem S. (2006). Annually reoccurring bacterial communities are predictable from ocean conditions. *Proc. Natl. Acad. Sci. U.S.A.* 103 13104–13109. 10.1073/pnas.0602399103 16938845 PMC1559760

[B36] Gasol J. Doval M. Pinhassi J. Calderón-Paz J. Guixa-Boixareu N. Vaqué D. (1998). Diel variations in bacterial heterotrophic production in the Northwestern Mediterranean Sea. *Mar. Ecol. Prog. Ser.* 164 125–133. 10.3354/meps164107

[B37] Gasol J. M. del Giorgio P. A. Duarte C. M. (1997). Biomass distribution in marine planktonic communities. *Limnol. Oceanogr.* 42 1353–1363. 10.4319/lo.1997.42.6.1353

[B38] Gilbert J. A. Steele J. A. Caporaso J. G. Steinbrück L. Reeder J. Temperton B. (2012). Defining seasonal marine microbial community dynamics. *ISME J.* 6 298–308. 10.1038/ismej.2011.107 21850055 PMC3260500

[B39] Giorgio P. A. D. Bird D. F. Prairie Y. T. Planas D. (1996). Flow cytometric determination of bacterial abundance in lake plankton with the green nucleic acid stain SYTO 13. *Limnol. Oceanogr.* 41 783–789. 10.4319/lo.1996.41.4.0783

[B40] Gómez-Consarnau L. Lindh M. V. Gasol J. M. Pinhassi J. (2012). Structuring of bacterioplankton communities by specific dissolved organic carbon compounds. *Environ. Microbiol.* 14 2361–2378. 10.1111/j.1462-2920.2012.02804.x 22697392

[B41] Granéli E. Wallström K. Larsson U. Granéli W. Elmgren R. (1990). Nutrient limitation of primary production in the Baltic Sea area. *AMBIO* 19 142–151. 11697250

[B42] Gunderson A. R. Armstrong E. J. Stillman J. H. (2016). Multiple stressors in a changing world: the need for an improved perspective on physiological responses to the dynamic marine environment. *Annu. Rev. Mar. Sci.* 8 357–378. 10.1146/annurev-marine-122414-033953 26359817

[B43] Haverkamp T. Schouten D. Doeleman M. Wollenzien U. Huisman J. Stal L. (2009). Colorful microdiversity of *Synechococcus* strains (picocyanobacteria) isolated from the Baltic Sea. *ISME J.* 3:397. 10.1038/ismej.2008.118 19052629

[B44] HELCOM. (2018). *Chlorophyll A. HELCOM Core Indicator Report.* Available at: http://www.helcom.fi/Core%20Indicators/Chlorophyll%20a%20HELCOM%20core%20indicator%202018.pdf. [accessed November 20 2018].

[B45] Hewson I. Steele J. A. Capone D. G. Fuhrman J. A. (2006). Temporal and spatial scales of variation in bacterioplankton assemblages of oligotrophic surface waters. *Mar. Ecol. Prog. Ser.* 311 67–77. 10.3354/meps311067

[B46] Ho T. Y. Scranton M. I Taylor G. T. Varela R. Thunell R. C. Muller-Karger F. (2002). Acetate cycling in the water column of the Cariaco Basin: seasonal and vertical variability and implication for carbon cycling. *Limnol. Oceanogr.* 47 1119–1128. 10.4319/lo.2002.47.4.1119

[B47] Hoppe H.-G. Giesenhagen H. C. Koppe R. Hansen H. P. Gocke K. (2013). Impact of change in climate and policy from 1988 to 2007 on environmental and microbial viariables at the time series station Boknis Eck, Baltic Sea. *Biogeosciences* 10 4529–4546. 10.5194/bg-10-4529-2013

[B48] Hugerth L. W. Larsson J. Alneberg J. Lindh M. V. Legrand C. Pinhassi J. (2015). Metagenome-assembled genomes uncover a global brackish microbiome. *Genome Biol.* 16:279. 10.1186/s13059-015-0834-7 26667648 PMC4699468

[B49] Huseby S. Wikner J. (2015). Bacterioplankton Growth. 2015 *HELCOM Baltic Sea Fact Sheets.* Available at: http://www.helcom.fi/baltic-sea-trends/environment-fact-sheets/ [accessed June 13 2017].

[B50] Jespersen A. M. Christoffersen K. (1987). Measurements of chlorophyll-a from phytoplankton using ethanol as extraction solvent. *Archiv. Hydrobiol.* 109 445–454.

[B51] Karl D. (2014). Microbially mediated transformations of phosphorus in the Sea: new views of an old cycle. *Annu. Rev. Mar. Sci.* 6 279–337. 10.1146/annurev-marine-010213-135046 24405427

[B52] Karl D. M. Lukas R. (1996). The Hawaii Ocean time-series (HOT) program: background, rationale and field implementation. *Deep Sea Res. II* 43 129–156. 10.1016/0967-0645(96)00005-7

[B53] Karlson A. M. Duberg J. Motwani N. H. Hogfors H. Klawonn I. Ploug H. (2015). Nitrogen fixation by cyanobacteria stimulates production in Baltic food webs. *AMBIO* 44 413–426. 10.1007/s13280-015-0660-x 26022324 PMC4447702

[B54] Klais R. Tamminen T. Kremp A. Spilling K. Olli K. (2011). Decadal-scale changes of dinoflagellates and diatoms in the anomalous Baltic Sea spring bloom. *PloS One* 6:e21567. 10.1371/journal.pone.0021567 21747911 PMC3126836

[B55] Komárek J. Kopecký J. Cepák V. (1999). Generic characters of the simplest cyanoprokaryotes *Cyanobium*, Cyanobacterium and *Synechococcus*. *Cryptogam. Algol.* 20 209–222. 10.1016/S0181-1568(99)80015-4

[B56] Kuosa H. (1991). Picoplanktonic algae in the northern Baltic Sea: seasonal dynamis and flagellate grazing. *Mar. Ecol. Prog. Ser.* 73 269–276. 10.3354/meps073269

[B57] Kuosa H. Kivi K. (1989). Bacteria and heterotrophic flagellates in the pelagic carbon cycle in the northern Baltic Sea. *Mar. Ecol. Prog. Ser.* 53 93–100. 10.3354/meps053093

[B58] Legrand C. Fridolfsson E. Bertos-Fortis M. Lindehoff E. Larsson P. Pinhassi J. (2015). Interannual variability of phyto-bacterioplankton biomass and production in coastal and offshore waters of the Baltic Sea. *AMBIO* 44 S427–S438. 10.1007/s13280-015-0662-8 26022325 PMC4447688

[B59] Lindh M. V. (2014). *Bacterioplankton Population Dynamics in a Changing Ocean.* Sweden: Linnaeus University Press.

[B60] Lindh M. V. Sjöstedt J. Andersson A. F. Baltar F. Hugerth L. W. Lundin D. (2015). Disentangling seasonal bacterioplankton population dynamics by high frequency sampling. *Environ. Microbiol.* 17 2459–2476. 10.1111/1462-2920.12720 25403576

[B61] Lindh M. V. Sjöstedt J. Ekstam B. Casini M. Lundin D. Hugerth L. W. (2017). Metapopulation theory identifies biogeographical patterns among core and satellite marine bacteria scaling from tens to thousands of kilometers. *Environ. Microbiol.* 19 1222–1236. 10.1111/1462-2920.13650 28028880

[B62] Liu Z. Liu S. (2016). High phosphate concentrations accelerate bacterial peptide decomposition in hypoxic bottom waters of the northern Gulf of Mexico. *Eviron. Sci. Technol.* 50 676–684. 10.1021/acs.est.5b03039 26650147

[B63] Martin-Platero A. Cleary B. Kauffman K. Preheim S. McGillicuddy D. J. Alm E. (2018). High resolution time series reveals cohesive but short-lived communities in coastal plankton. *Nat. Commun.* 9:266. 10.1038/s41467-017-02571-4 29348571 PMC5773528

[B64] Mayali X. Palenik B. Burton R. (2010). Dynamics of marine bacterial and phytoplankton populations using multipley liquid bead array technology. *Environ. Microbiol.* 12 975–989. 10.1111/j.1462-2920.2004.02142.x 20105218

[B65] Mazur-Marzec H. Sutryk K. Hebel A. Hohlfeld N. Pietrasik A. Błaszczyk A. (2015). Nodularia spumigena peptides—accumulation and effect on aquatic invertebrates. *Toxins* 7 4404–4420. 10.3390/toxins7114404 26529012 PMC4663510

[B66] Needham D. M. Chow C.-E. T. Cram J. A. Sachdeva R. Parada A. Fuhrman J. A. (2013). Short-term observations of marine bacterial and viral communities: patterns, connections and resilience. *ISME J.* 7 1274–1285. 10.1038/ismej.2013.19 23446831 PMC3695287

[B67] Needham D. M. Fuhrman J. A. (2016). Pronounced daily succession of phytoplankton, archaea and bacteria following a spring bloom. *Nat. Microbiol.* 1:16005. 10.1038/nmicrobiol.2016.5 27572439

[B68] Not F. del Campo J. Balagué V. de Vargas C. Massana R. (2009). New insights into the diversity of marine picoeukaryotes. *PLoS One* 4:e7143. 10.1371/journal.pone.0007143 19787059 PMC2747013

[B69] Obernosterer I. Reitner B. Herndl G. J. (1999). Contrasting effects of solar radiation on dissolved organic matter and its bioavailability to marine bacterioplankton. *Limnol. Oceanogr.* 44 1645–1654. 10.4319/lo.1999.44.7.1645

[B70] Oksanen J. Kindt R. Legendre P. O’Hara B. Stevens M. H. H. Oksanen M. J. (2007). *Vegan: Community Ecology Package. R package version1.17-5.*

[B71] Olenina I. Hajdu S. Edler L. Andersson A. Wasmund N. Busch S. (2006). *Biovolumes and Size-Classes of Phytoplankton in the Baltic Sea.* Helsinki: HELCOM Baltic Sea Environment Proceedings 144.

[B72] Ottesen E. Marin R. Preston C. Young C. Ryan J. Scholin C. (2011). Metatranscriptomic analysis of autonomously collected and preserved marine bacterioplankton. *ISME J.* 5 1881–1895. 10.1038/ismej.2011.70 21716310 PMC3223310

[B73] Ottesen E. Young C. Gifford S. Eppley J. Marin R. I. Schuster S. (2014). Multispecies diel transcriptional oscillations in open ocean heterotrophic bacterial assemblages. *Science* 345 207–212. 10.1126/science.1252476 25013074

[B74] Paerl R. Sundh J. Tan D. Svenningsen S. Hylander S. Pinhassi J. (2018). Prevalent reliance of bacterioplankton on exogenous vitamin B1 and precursor availability. *Proc. Natl. Acad. Sci. U.S.A.* 115 E10447–E10456. 10.1073/pnas.1806425115 30322929 PMC6217396

[B75] Pettersson H. Lindow H. Schrader D. (2012). *Wave Climate in the Baltic Sea 2011. HELCOM Baltic Sea Environment Fact Sheets.* Available at: http://www.helcom.fi/baltic-sea-trends/environment-fact-sheets/

[B76] Pinhassi J. Azam F. Hemphälä J. Long R. A. Martinez J. Zweifel U. L. (1999). Coupling between bacterioplankton species composition, population dynamics, and organic matter degradation. *Aquat. Microb. Ecol.* 17:13 10.3354/ame017013

[B77] Pinhassi J. Gómez-Consarnau L. Alonso-Sáez L. Sala M. M. Vidal M. Pedrós-Alio C. (2006). Seasonal changes in bacterioplankton nutrient limitation and their effects on bacterial community composition in the NW Mediterranean Sea. *Aquat. Microb. Ecol.* 44 241–252. 10.3354/ame044241

[B78] Pinhassi J. Hagström Å. (2000). Seasonal succession in marine bacterioplankton. *Aquat. Microb. Ecol.* 21 245–256. 10.3354/ame021245

[B79] Pinhassi J. Sala M. M. Havskum H. Peters F. Guadayol Ò. Malits A. (2004). Changes in bacterioplankton composition under different phytoplankton regimes. *Appl. Environ. Microbiol.* 70 6753–6766. 10.1128/AEM.70.11.6753-6766.2004 15528542 PMC525254

[B80] Piwosz K. Pernthaler J. (2010). Seasonal population dynamics and trophic role of planktonic nanoflagellates in coastal surface waters of the Southern Baltic Sea. *Environ. Microbiol.* 12 364–377. 10.1111/j.1462-2920.2009.02074.x 19799618

[B81] Rich J. H. Ducklow H. W. Kirchman D. L. (1996). Concentrations and uptake of neutral monosaccharides along 14 W in the equatorial Pacific: contribution of glucose to heterotrophic bacterial activity and the DOM flux. *Limnol. Oceanogr.* 41 595–604. 10.4319/lo.1996.41.4.0595

[B82] Samuelsson K. Berglund J. Andersson A. (2006). Factors structuring the heterotrophic flagellate and ciliate community along a brackish water primary production gradient. *J. Plankton Res.* 28 345–359. 10.1093/plankt/fbi118

[B83] Sarmento H. Gasol J. M. (2012). Use of phytoplankton-derived dissolved organic carbon by different types of bacterioplankton. *Environ. Microbiol.* 14 2348–2360. 10.1111/j.1462-2920.2012.02787.x 22639946

[B84] Seidel M. Manecki M. Herlemann D. P. Deutsch B. Schulz-Bull D. Jürgens K. (2017). Composition and transformation of dissolved organic matter in the Baltic Sea. *Front. Earth Sci.* 5:31 10.3389/feart.2017.00031

[B85] Siegel H. Gerth M. (2012). *Sea Surface Temperature in the Baltic Sea 2011. HELCOM Baltic Sea Environment Fact Sheets.* Available at: http://www.helcom.fi/baltic-sea-trends/environment-fact-sheets/

[B86] Simó R. Archer S. D. Pedrós-Alió C. Gilpin L. Stelfox-Widdicombe C. E. (2002). Coupled dynamics of dimethylsulfoniopropionate and dimethylsulfide cycling and the microbial food web in surface waters of the North Atlantic. *Limnol. Oceanogr.* 47 53–61. 10.4319/lo.2002.47.1.0053

[B87] Simon M. Azam F. (1989). Protein content and protein synthesis rates of planktonic marine bacteria. *Mar. Ecol. Prog. Ser.* 51 201–213. 10.3354/meps051201 11679347

[B88] Skoog A. Biddanda B. Benner R. (1999). Bacterial utilization of dissolved glucose in the upper water column of the Gulf of Mexico. *Limnol. Oceanogr.* 44 1625–1633. 10.3389/fmicb.2013.00318

[B89] Smith D. C. Azam F. (1992). A simple, economical method for measuring bacterial protein synthesis rates in seawater using 3H-leucine. *Mar. Microb. Food Webs* 6 107–114.

[B90] Smriga S. Fernandez V. I. Mitchell J. G. Stocker R. (2016). Chemotaxis toward phytoplankton drives organic matter partitioning among marine bacteria. *Proc. Natl. Acad. Sci. U.S.A.* 113 1576–1581. 10.1073/pnas.1512307113 26802122 PMC4760798

[B91] Suikkanen S. Laamnen M. Huttunen M. (2007). Long-term changes in summer phytoplankton communities of the open northern Baltic Sea. *Estuar. Coast. Shelf Sci.* 71 580–592. 10.1016/j.ecss.2006.09.004 18488550

[B92] Suttle C. A. Chan A. M. Fuhrman J. A. (1991). Dissolved free amino acids in the Sargasso Sea: uptake and respiration rates, turnover times, and concentrations. *Mar. Ecol. Prog. Ser.* 70 189–199. 10.3354/meps070189

[B93] Tamelander T. Heiskanen A.-S. (2004). Effects of spring bloom phytoplankton dynamics and hydrography on the composition of settling material in the coastal northern Baltic Sea. *J. Mar. Syst.* 52 217–234. 10.1016/j.jmarsys.2004.02.001

[B94] Teeling H. Fuchs B. M. Becher D. Klockow C. Gardebrecht A. Bennke C. M. (2012). Substrate-controlled succession of marine bacterioplankton populations induced by a phytoplankton bloom. *Science* 336 608–611. 10.1126/science.1218344 22556258

[B95] Teeling H. Fuchs B. M. Bennke C. M. Krüger K. Chafee M. Kappelmann L. (2016). Recurring patterns in bacterioplankton dynamics during coastal spring algae blooms. *eLife* 5:e11888. 10.7554/eLife.11888 27054497 PMC4829426

[B96] Thamm R. Schernewski G. Wasmund N. Neumann T. (2004). *Spatial Phytoplankton Pattern in the Baltic Sea.* Leiden: EUCC – The Coastal Union.

[B97] Tiselius P. Belgrano A. Andersson L. Lindahl O. (2015). Primary productivity in a coastal ecosystem: a trophic perspective on a long-term time series. *J. Plankton Res.* 38 1092–1102. 10.1093/plankt/fbv094

[B98] Traving S. Rowe O. Jakobsen N. Sørensen H. Dinasquet J. Stedtmon C. (2017). The Effect of increased loads of dissolved organic matter on estuarine microbial community composition and function. *Front. Microbiol.* 8:351. 10.3389/fmicb.2017.00351 28337180 PMC5343018

[B99] Utermöhl H. (1958). Zur vervollkommnung der quantitativen phytoplankton-methodik. *Mitt. Int. Verein. Theor. Angew. Limnol.* 9 1–38. 10.1080/05384680.1958.11904091

[B100] Utermöhl V. H. (1931). Neue wege in der quantitativen erfassung des planktons. (Mit besondere berücksichtigung des ultraplanktons). *Verh. Int. Verein. Theor. Angew. Limnol.* 5 567–595. 10.1080/03680770.1931.11898492

[B101] Vahtera E. Conley D. J. Gustafsson B. G. Kuosa H. Pitkänen H. Savchuk O. P. (2007). Internal ecosystem feedbacks enhance nitrogen-fixing cyanobacteria blooms and complicate management in the Baltic Sea. *AMBIO* 36 186–194. 10.1579/0044-7447(2007)36[186:IEFENC]2.0.CO;2 17520933

[B102] Valderrama J. C. (1995). “Methods of nutrient analysis,” in *Manual on Harmful Marine Microalgae. IOC Manuals and Guides* eds Hallagraeff G. M. Anderson D. M. Cembella A. D. (Paris: UNESCO) 251–268.

[B103] Vila-Costa M. Simó R. Harada H. Gasol J. M. Slezak D. Kiene R. P. (2006). Dimethylsulfoniopropionate uptake by marine phytoplankton. *Science* 314 652–654. 10.1126/science.1131043 17068265

[B104] Ward C. S. Yung C.-M. Davis K. M. Blinebry S. K. Williams T. C. Johnson Z. I. (2017). Annual community patterns are driven by seasonal switching between closely related marine bacteria. *ISME J.* 11 1412–1422. 10.1038/ismej.2017.4 28234350 PMC5437356

[B105] Wasmund N. Tuimala J. Suikkanen S. Vandepitte L. Kraberg A. (2011). Long-term trends in phytoplankton composition in the western and central Baltic Sea. *J. Mar. Syst.* 87 145–159. 10.1016/j.jmarsys.2011.03.010

[B106] Wasmund N. Uhlig S. (2003). Phytoplankton trends in the Baltic Sea. *ICES J. Mar. Sci.* 60 177–186. 10.1016/S1054-3139(02)00280-1

[B107] Wickham H. (2009). *ggplot2: Elegant Graphics for Data Analysis.* New York, NY: Springer 10.1007/978-0-387-98141-3

[B108] Wikner J. Andersson A. (2012). Increased freshwater discharge shifts the trophic balance in the coastal zone of the northern Baltic Sea. *Glob. Change Biol.* 18 2509–2519. 10.1111/j.1365-2486.2012.02718.x

[B109] Worden A. Z. Not F. (2008). “Ecology and diversity of picoeukaryotes,” in *Microbial Ecology of the Oceans* 2nd Edn eds Gasol J. M. Kirchman D. L. (Hoboken, NJ: John Wiley & Sons) 159–205. 10.1002/9780470281840.ch6

[B110] Xing P. Hahnke R. Unfried F. Markert S. Huang S. Barbeyron T. (2015). Niches of two polysaccharide-degrading Polaribacter isolates from the North Sea during a spring diatom bloom. *ISME J.* 9 1410–1422. 10.1038/ismej.2014.225 25478683 PMC4438327

[B111] Yang J. Wu W. Chung C. Chiang K. Gong G. Hsieh C. (2018). Predator and prey biodiversity relationship and its consequences on marine ecosystem functioning—interplay between nanoflagellates and bacterioplankton. *ISME J.* 12:1532. 10.1038/s41396-018-0111-3 29703955 PMC5956075

[B112] Zweifel U.-L. Norrman B. Hagström Å. (1993). Consumption of dissolved organic carbon by marine bacteria and demand for inorganic nutrients. *Mar. Ecol. Prog. Ser.* 101 23–32. 10.3354/meps101023

[B113] Zweifel U. L. Wikner J. Hagström Å. Lundberg E. Norrman B. (1995). Dynamics of dissolved organic carbon in a coastal ecosystem. *Limnol. Oceanogr.* 40 299–305. 10.4319/lo.1995.40.2.0299

