# Supplementary figures and images for: High Frequency Multi-Year Variability in Baltic Sea Microbial Plankton Stocks and Activities
Source: Front Microbiol. 2019 Jan 17;9:3296. doi: 10.3389/fmicb.2018.03296 (PMC6345115; doi:10.3389/fmicb.2018.03296)

Suppl. Figure 1

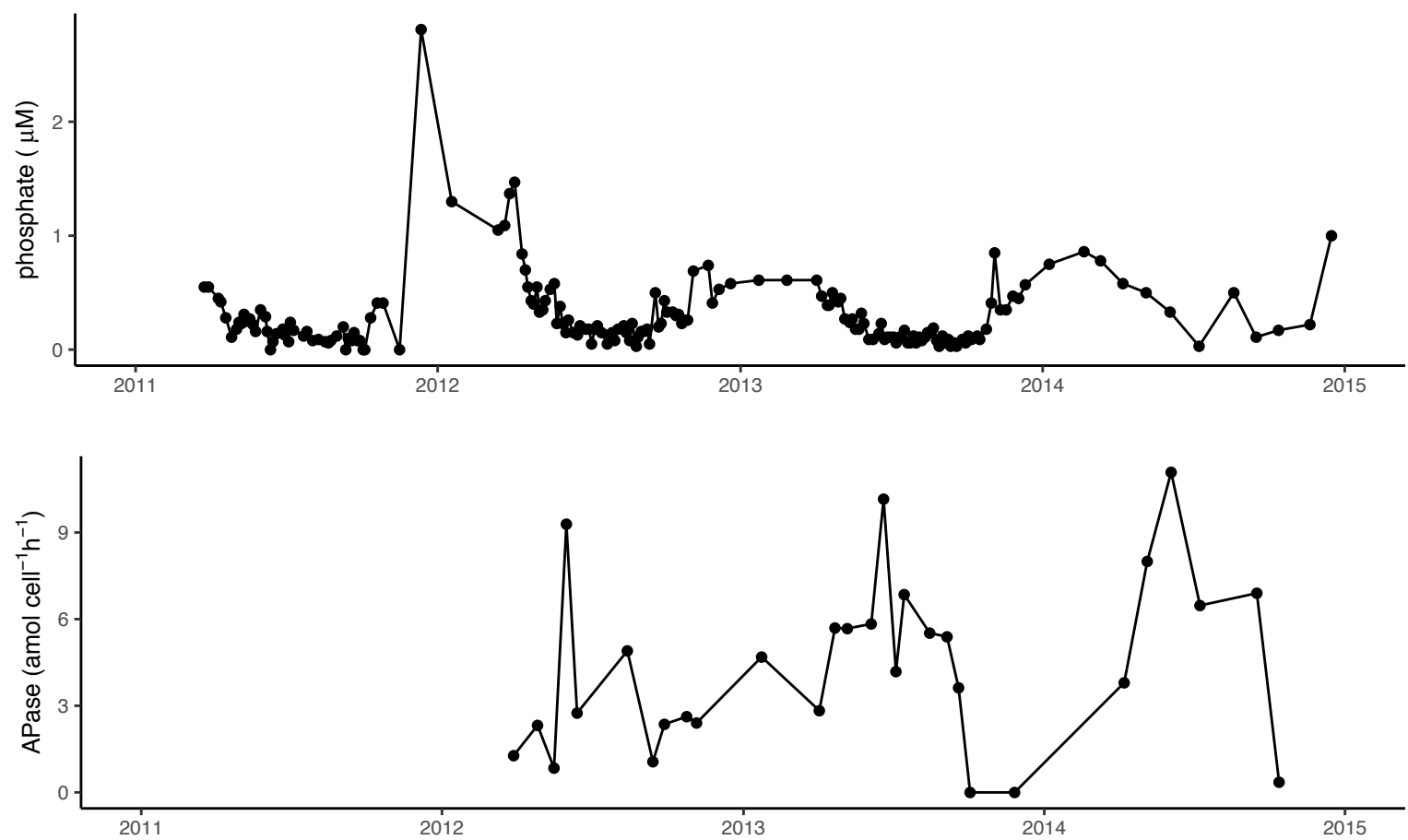

Supplement: FIGURE S1 — Linegraph of (A) phosphate and (B) APase activity over time. [file Image_1.pdf]

Supplementary Figure 2

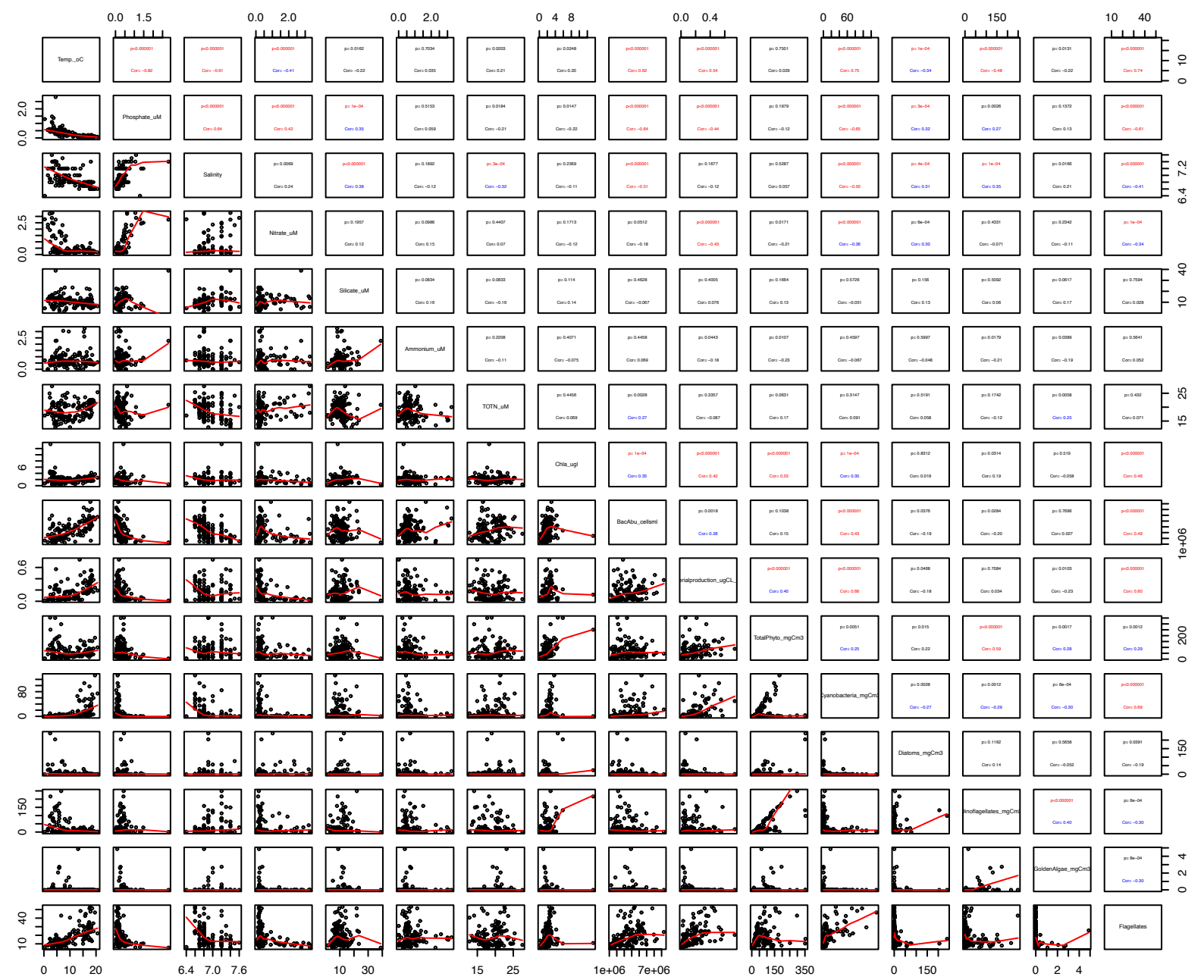

Supplement: FIGURE S2 — Spearman’s rank correlation test results of abiotic and biotic variables against each other 2011–2014, excluding the variable DOC. Spearman’s rho is indicated as “Cor,” p-values are indicated as “p.” Colors denote significance thresholds: red p-values denote significant values after Bonferroni correction ∗p > 0.05, red Cor denote Spearman’s rho values > 0.45, blue denotes Spearman’s rho values > 0.25. [file Image_2.pdf]

Supplementary Figure 3

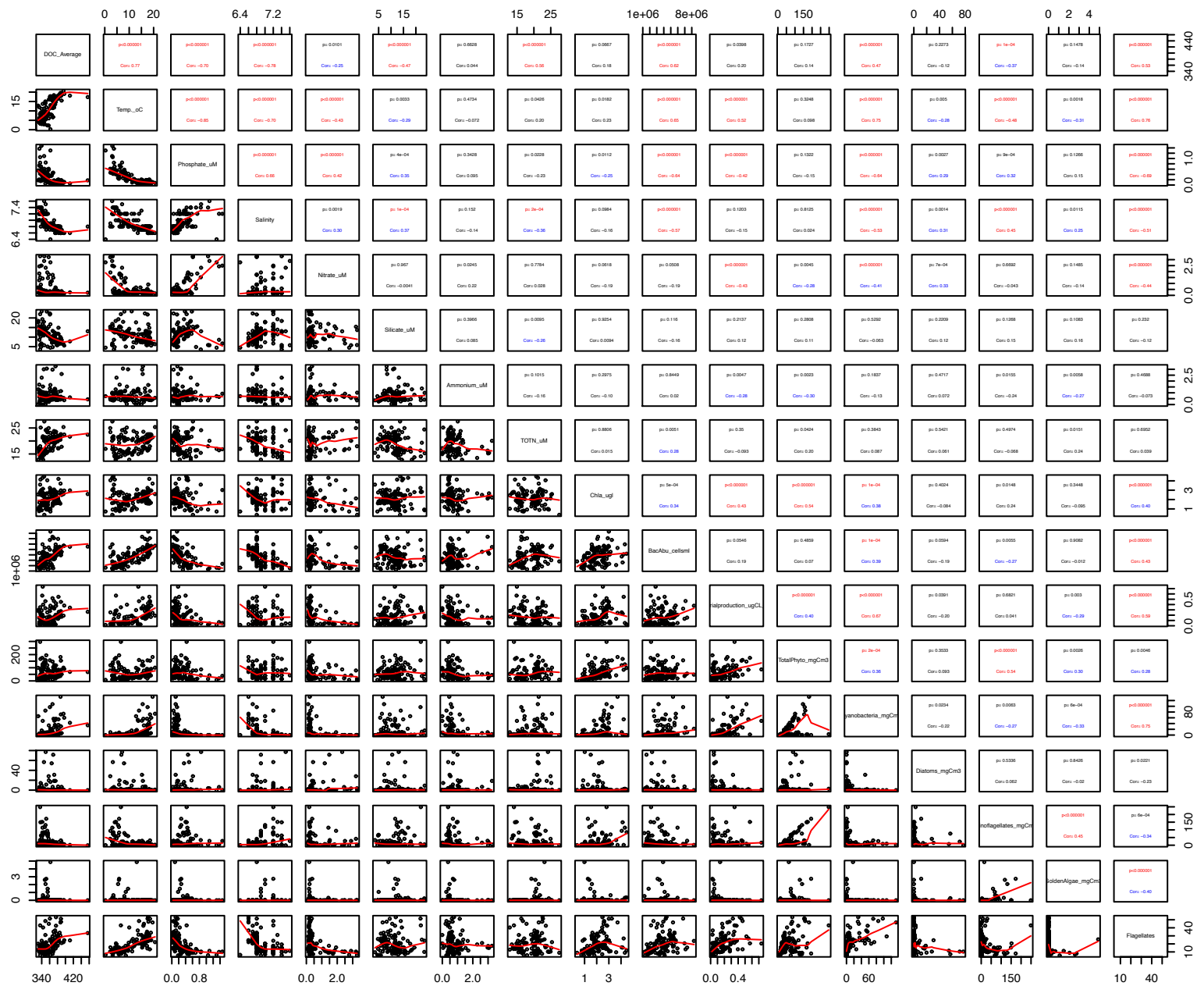

Supplement: FIGURE S3 — Spearman’s rank correlation test results of abiotic and biotic variables against each other including DOC values but excluding 2011 data. Spearman’s rho is indicated as “Cor,” p-values are indicated as “p.” Colors denote significance thresholds: red p-values denote significant values after Bonferroni correction ∗p > 0.05, red Cor denote Spearman’s rho values > 0.45, blue denotes Spearman’s rho values > 0.25. [file Image_3.pdf]

Supplementary Figure 4

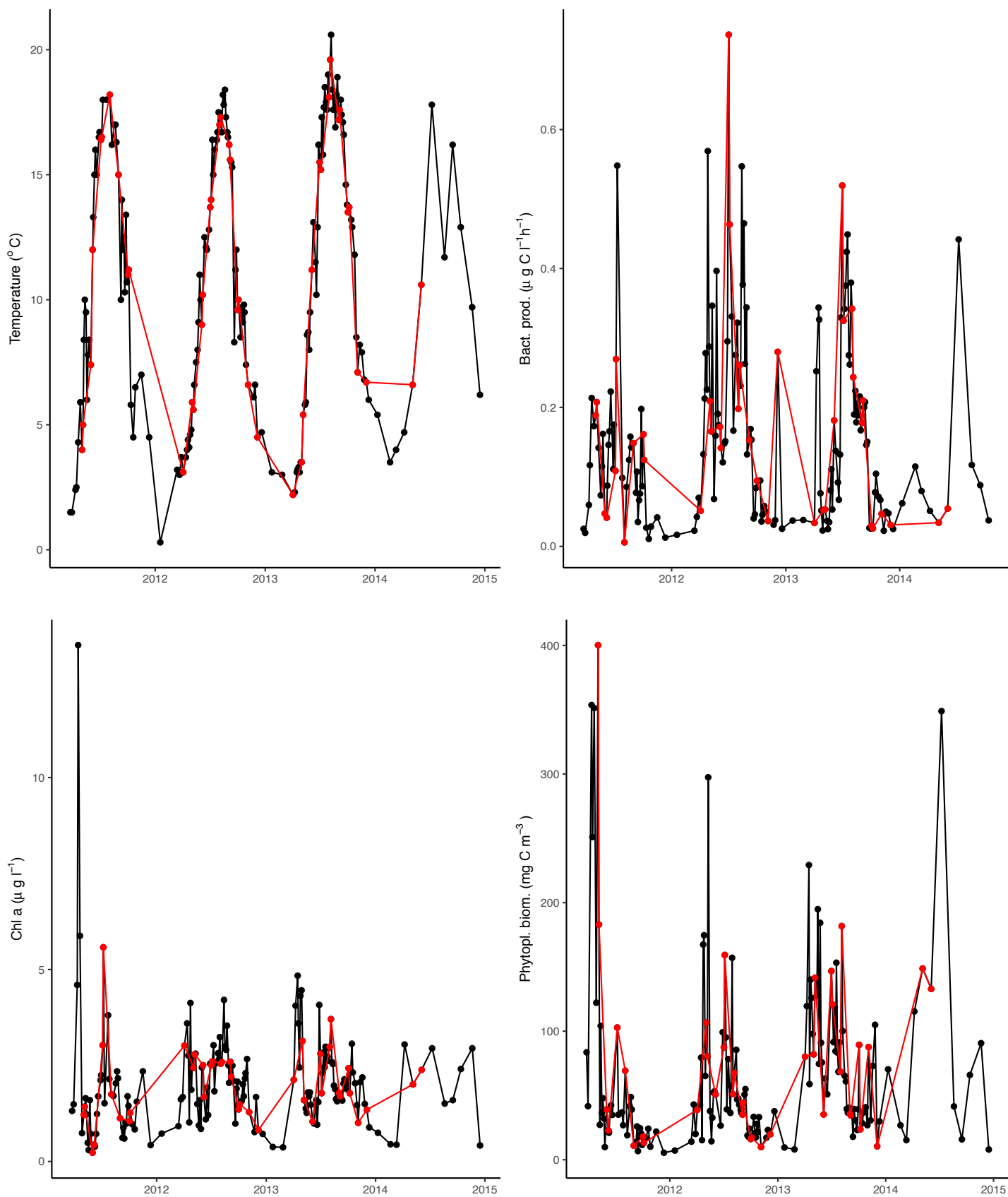

Supplement: FIGURE S4 — Subsampled data during 2011–2014. All data points that were sampled during the first week of the month (days 1–7) are labeled in red, underlying black points indicate all measured data for temperature (°C), bacterial heterotrophic production (μg C l-1 h-1), Chl a (μg l-1), and phytoplankton biomass (mgC m-3). [file Image_4.pdf]

Supplementary Figure 5

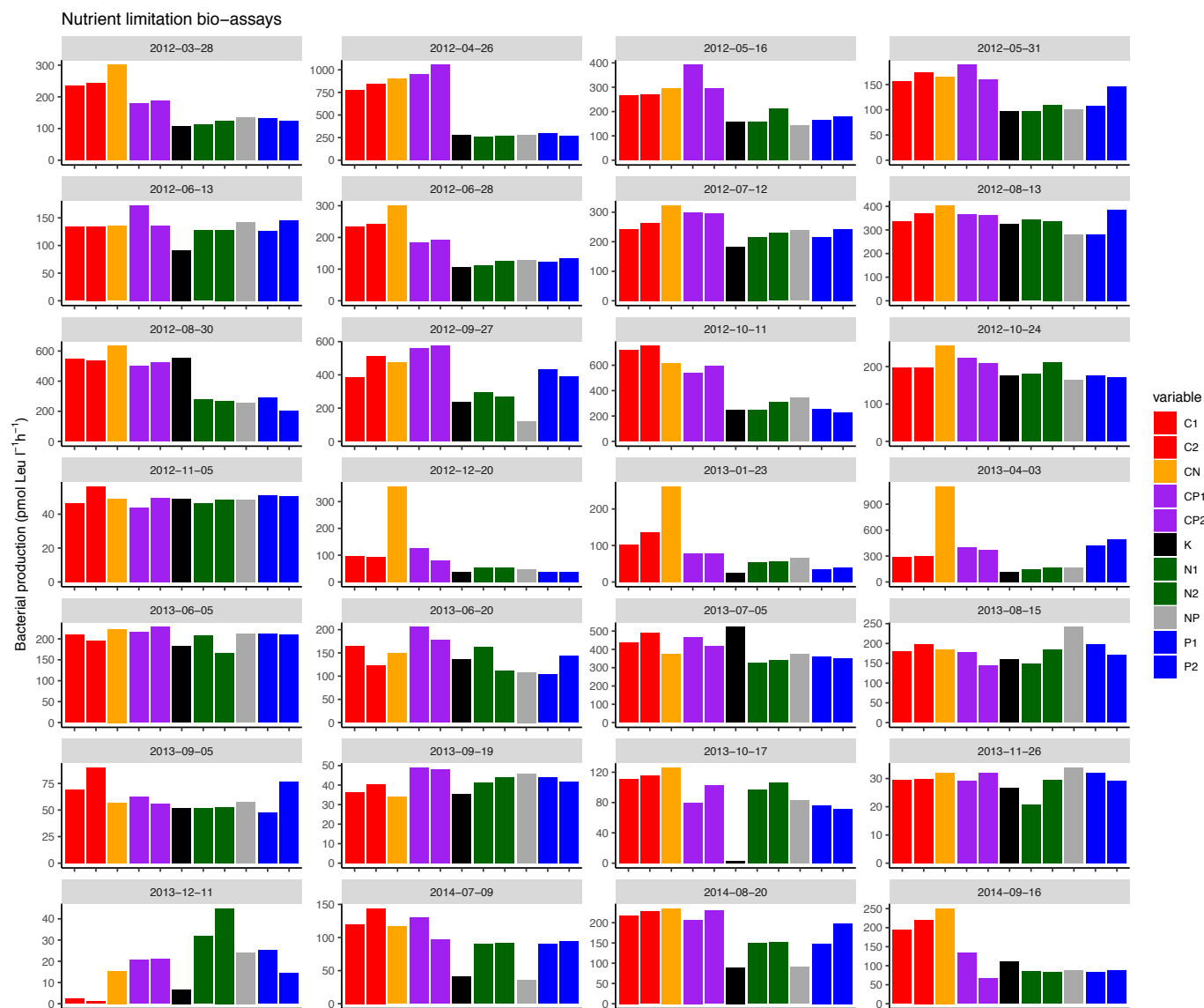

Supplement: FIGURE S5 — Nutrient limitation assays during 2012–2014. Displayed are the bacterial heterotrophic production estimates (pmol Leu l-1 h-1) of single or combined nutrient addition assays after 24 h incubation (biological replicates 1 or 2, respectively): N treatments (ammonium), P treatments (phosphate), and C treatments (glucose). Error bars indicate standard deviation of technical triplicates. [file Image_5.pdf]

Supplementary Figure 6

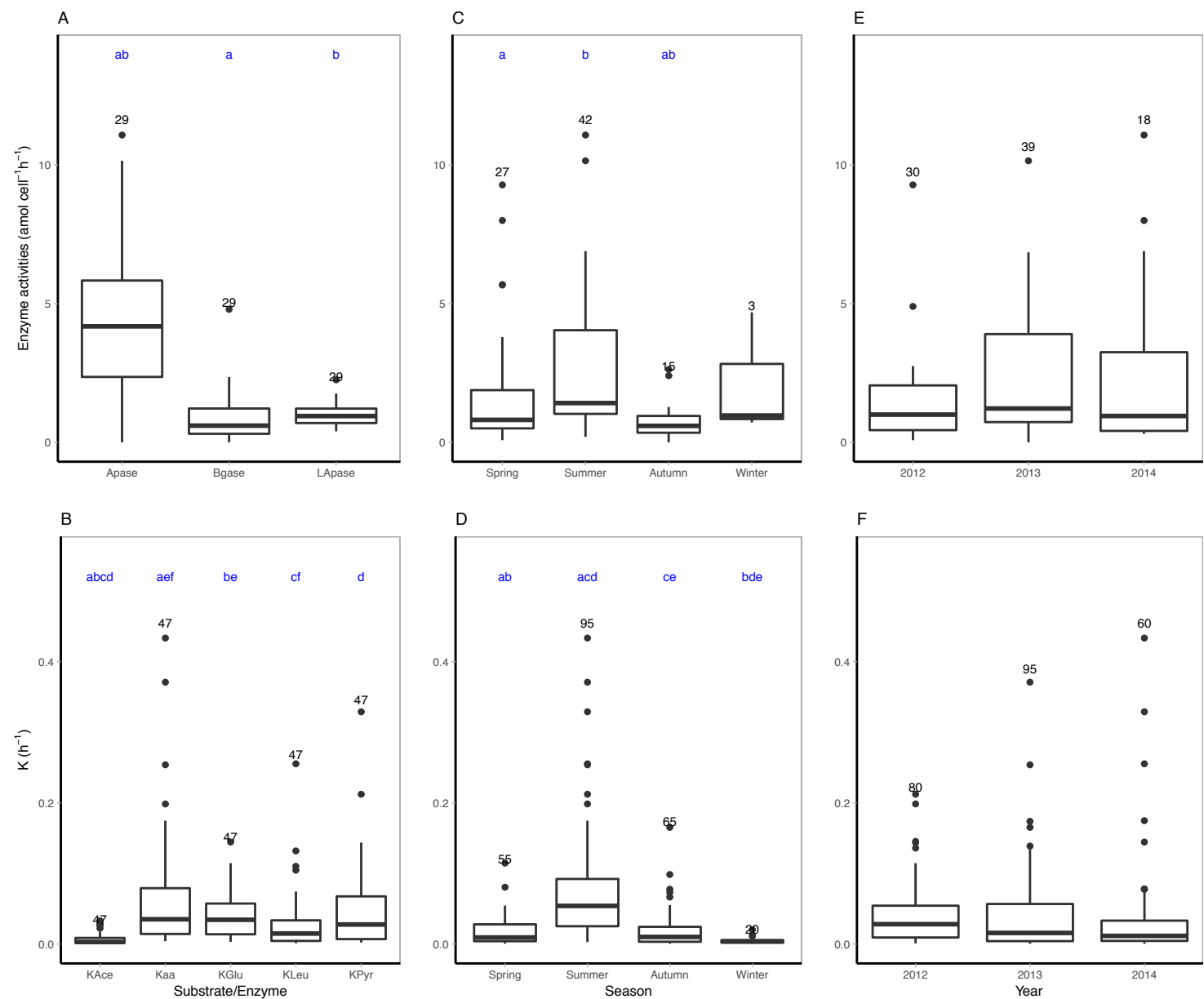

Supplement: FIGURE S6 — Boxplots of enzyme activities and substrate uptake rate constants, grouped by season, year, or enzyme/substrate. Enzyme activities normalized to bacterial abundances (amol cell-1 h-1) grouped by (A) enzyme, (C) season, and (E) year. APase denotes alkaline phosphatase, BGase denotes β-glucosidase, and LApase denotes leucine aminopeptidase. Substrate uptake rate constants K (h-1) grouped by (B) substrate, (D) season, and (F) year; acetate (KAce), amino acids (Kaa), glucose (KGlu), leucine (KLeu), and pyruvate (KPyr). The number of measurements per group is indicated above the boxplot and includes biological replicates as separate data points (#n). Letters above the boxplots indicate significant differences between seasons, years, or substrates/nutrients/enzymes (Monte Carlo simulation randomization test considered significantly different when ∗p < 0.05). [file Image_6.pdf]

## Supplementary Figure 7

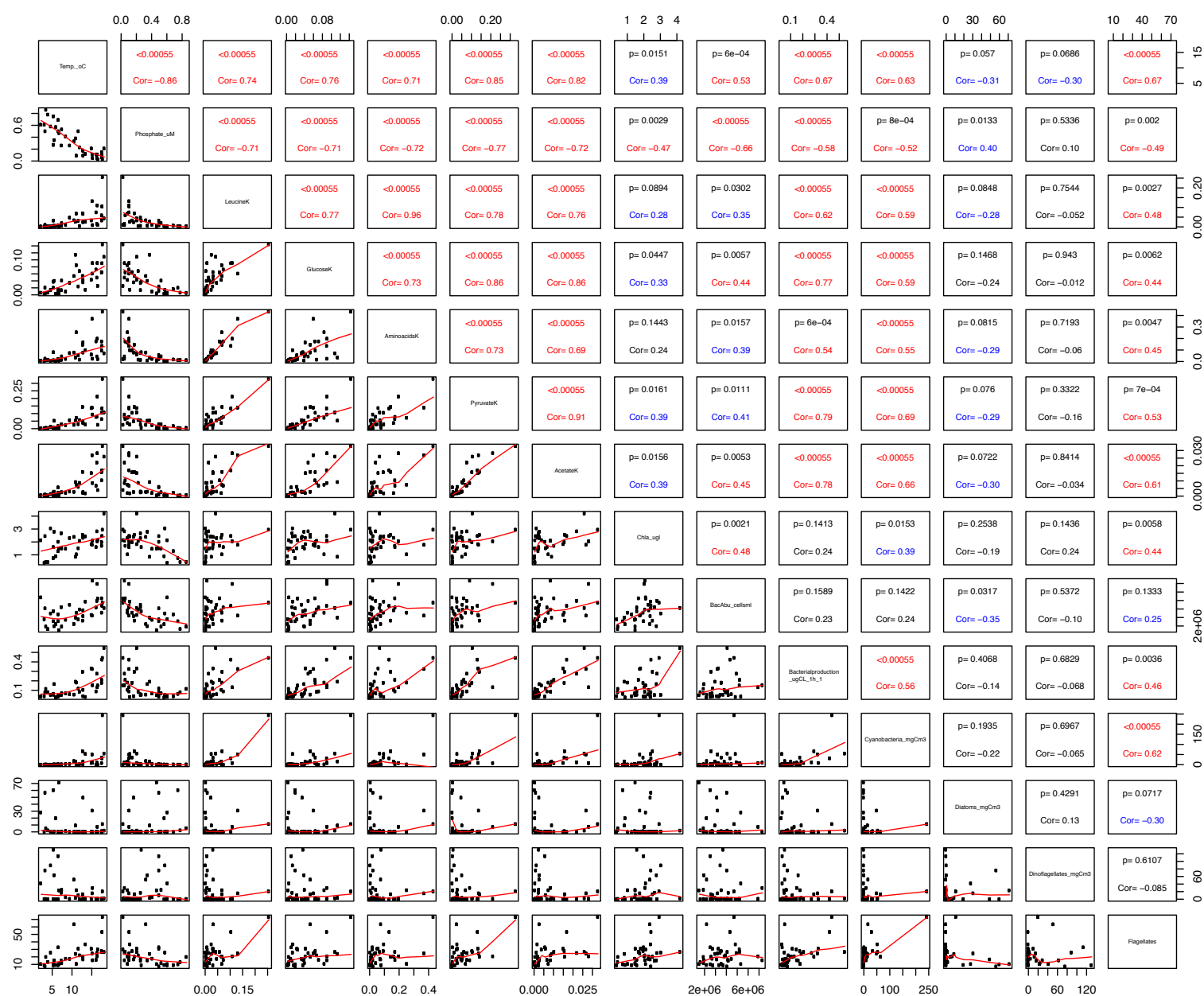

Supplement: FIGURE S7 — Spearman’s rank correlation test results of substrate uptake rate constants and biologically relevant variables excluding DOC. Spearman’s rho is indicated as “Cor,” p-values are indicated as “p.” Colors denote significance thresholds: red p-values denote significant values after Bonferroni correction ∗p > 0.05, red Cor denote Spearman’s rho values > 0.45, blue denotes Spearman’s rho values > 0.25. [file Image_7.pdf]

Supplementary Figure 8

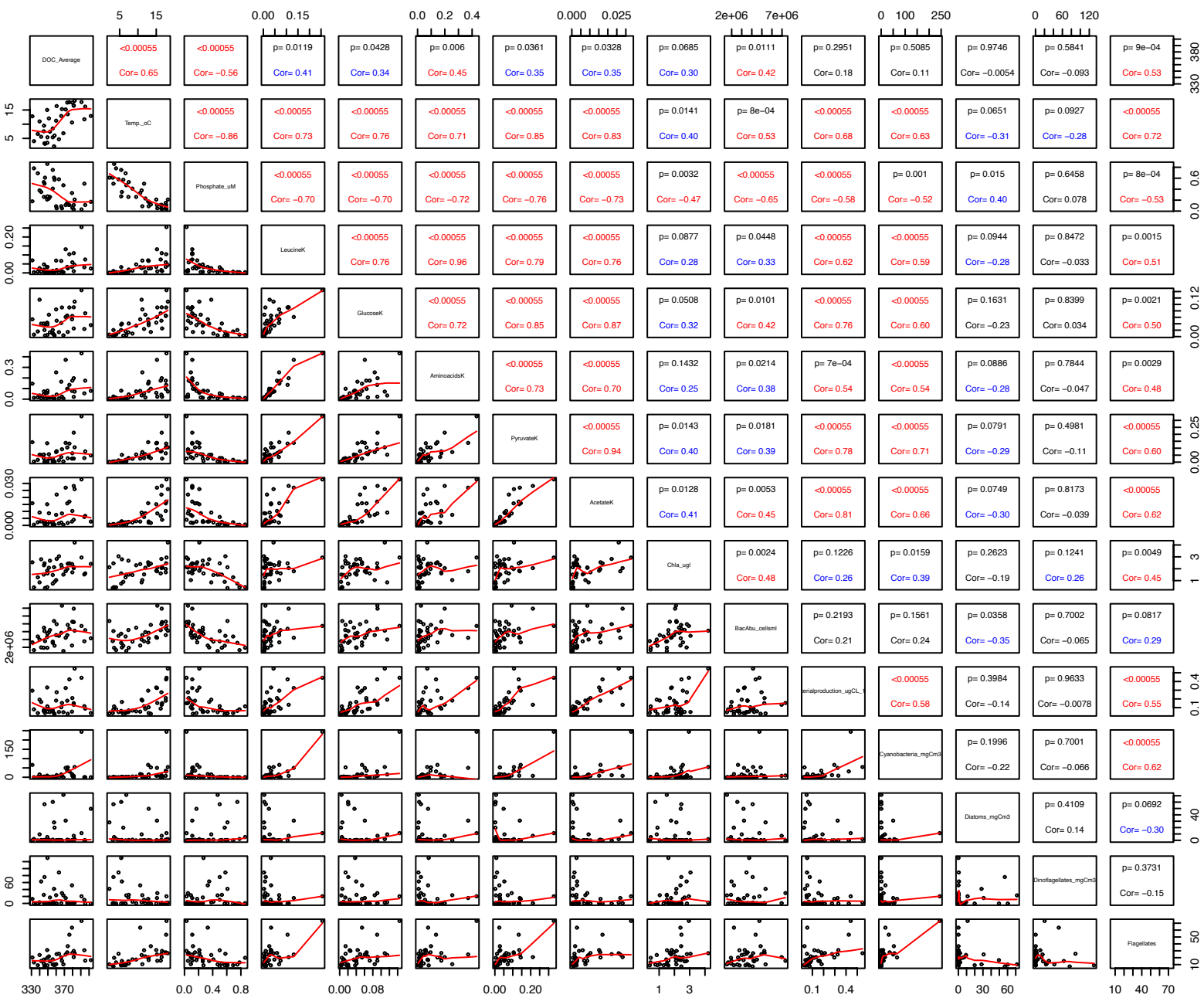

Supplement: FIGURE S8 — Spearman’s rank correlation test results of substrate uptake rate constants and biologically relevant variables including DOC. Spearman’s rho is indicated as “Cor”, p-values are indicated as “p”. Colors denote significance thresholds: red p-values denote significant values after Bonferroni correction ∗p > 0.05, red Cor denote Spearman’s rho values > 0.45, blue denotes Spearman’s rho values > 0.25. [file Image_8.pdf]

Supplementary Figure 9

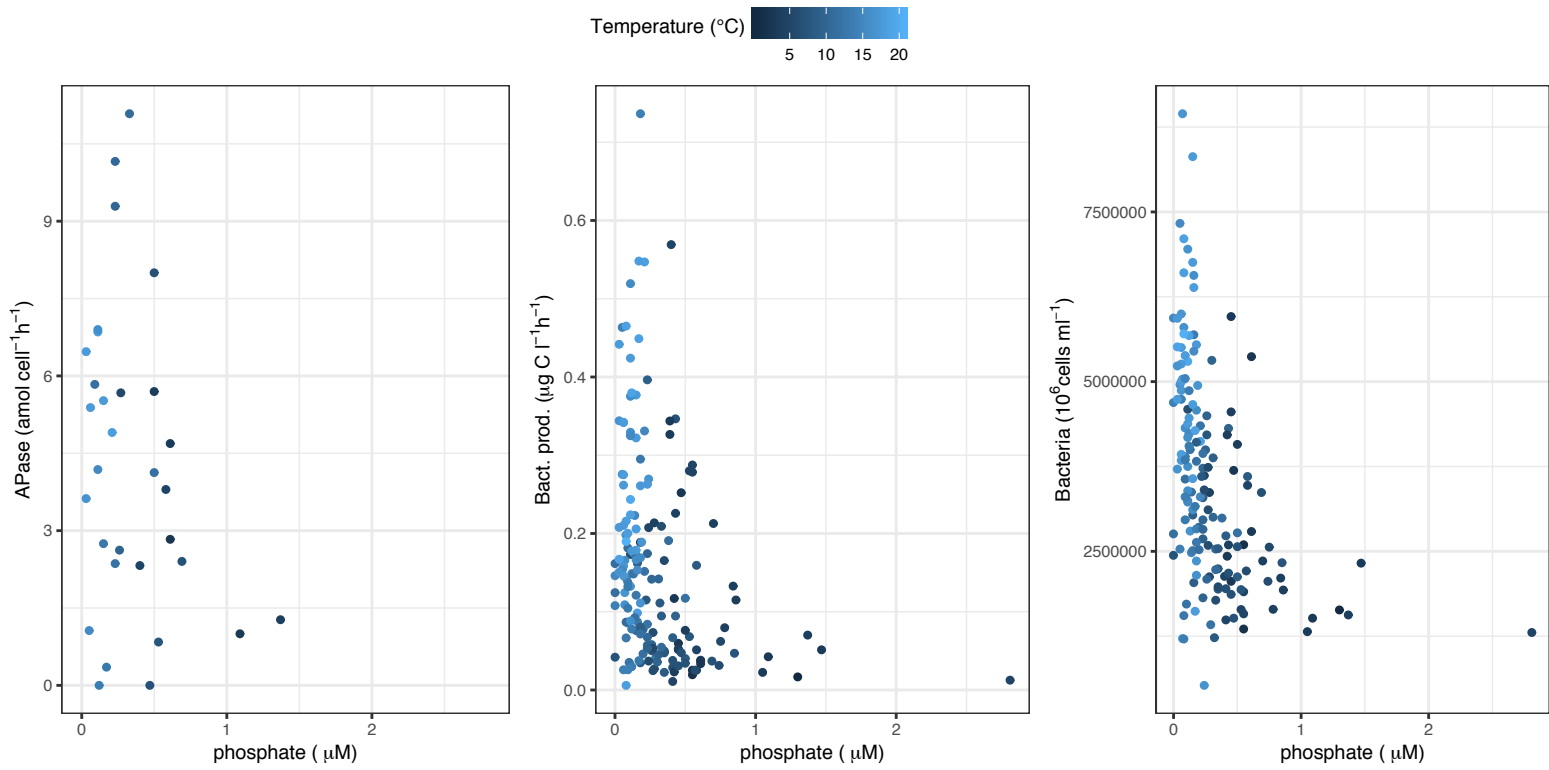

Supplement: FIGURE S9 — Graph of bacterial productivities and abundances plotted against phosphate concentrations during 2011–2014. (A) APase, (B) bacterial production, and (C) cell abundances. The color gradients denote temperatures at time of sampling. [file Image_9.pdf]
