# Supplementary material for: High Frequency Multi-Year Variability in Baltic Sea Microbial Plankton Stocks and Activities
Source: Front Microbiol. 2019 Jan 17;9:3296. doi: 10.3389/fmicb.2018.03296 (PMC6345115; doi:10.3389/fmicb.2018.03296)
Supplement: TABLE S1 — Chl a concentrations during summer time at LMO. Samples derive from 2m water depth and during the time between June and September. [file Table_1.pdf]

Supplementary Table 1. Chl a concentrations during summer time at LMO. Samples derive from 2m water depth and during the time between June and September.

Year	Mean Chl a (µg l^{-1})	sd	# samples
**2011**	1,677	1,162	26
**2012**	2,412	0,695	29
**2013**	2,158	0,739	32
**2014**	2,112	0,684	4
